# Deciphering Density Fluctuations in the Hydration Water of Brownian Nanoparticles via Upconversion Thermometry

**DOI:** 10.1021/acs.jpclett.4c00044

**Published:** 2024-02-29

**Authors:** Fernando E. Maturi, Ramon S. Raposo Filho, Carlos D. S. Brites, Jingyue Fan, Ruihua He, Bilin Zhuang, Xiaogang Liu, Luís D. Carlos

**Affiliations:** † Phantom-g, CICECO - Aveiro Institute of Materials, Department of Physics, University of Aveiro, 3810-193 Aveiro, Portugal; ‡ Institute of Chemistry, São Paulo State University (UNESP), 14800-060 Araraquara, SP, Brazil; § Department of Chemistry, National University of Singapore, Singapore 117543; ∥ Harvey Mudd College, 301 Platt Boulevard, Claremont, California 91711, United States

## Abstract

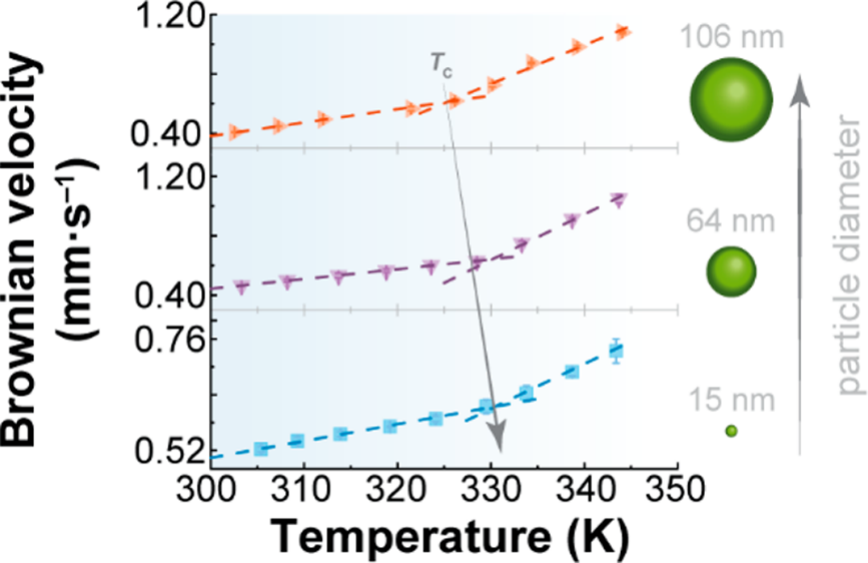

We investigate the intricate relationship among temperature, pH, and Brownian velocity in a range of differently sized upconversion nanoparticles (UCNPs) dispersed in water. These UCNPs, acting as nanorulers, offer insights into assessing the relative proportion of high-density and low-density liquid in the surrounding hydration water. The study reveals a size-dependent reduction in the onset temperature of liquid-water fluctuations, indicating an augmented presence of high-density liquid domains at the nanoparticle surfaces. The observed upper-temperature threshold is consistent with a hypothetical phase diagram of water, validating the two-state model. Moreover, an increase in pH disrupts the organization of water molecules, similar to external pressure effects, allowing simulation of the effects of temperature and pressure on hydrogen bonding networks. The findings underscore the significance of the surface of suspended nanoparticles for understanding high- to low-density liquid fluctuations and water behavior at charged interfaces.

Liquid water is the main constituent of the human body and covers a majority of the Earth’s surface. It plays a vital role in a myriad of biological, chemical, physical, geological, industrial, and environmental processes.^1−7^ In addition to its chemical properties as a solvent, proton transfer medium, and active component of reactions, the physical properties of water are also fundamentally relevant.^8^ Although water is the most commonly used liquid, its complex behavior under varying pressure and temperature conditions leads to numerous anomalies in its properties that differ significantly from those of other commonly used liquids. To date, more than 60 anomalous properties have been reported for water,^9−12^ including a minimum specific heat at 308 K, a negative thermal expansion coefficient below 277 K, and a minimum isothermal compressibility at 319 K.

The underlying reason for these anomalous characteristics is related to water’s remarkable ability to form strong and directional hydrogen bonds (H-bonds). As these hydrogen bonds are constantly breaking and re-forming on a picosecond time scale, fluctuations occur in the local structure of water, leading to the emergence of water motifs (or patches) with different densities.^11,12^ Today, two contrasting schools of thought seek to explain the anomalous properties of water by investigating its structural fluctuations. The continuous distribution models of water propose a homogeneous structural distribution caused by thermal fluctuations.^13−15^ The two-state model, on the contrary, argues that the anomalies stem from the coexistence of two distinct preferred local structural arrangements of water molecules that have different physical properties (e.g., the density differs by ∼20%^4^): a low-density liquid (LDL) and a high-density liquid (HDL). These local structures become increasingly well-defined upon supercooling and begin to contribute around the compressibility minimum.^4,8,11,16^ While LDL is an open tetrahedral configuration with predominantly low-energy H-bonds, HDL is a network with shorter and highly disordered H-bonds.^16−22^

This two-state scenario appears today to be very likely, as both theoretical^23−28^ and experimental results^29−40^ support the existence of a liquid–liquid critical point located in the supercooled liquid region of the water phase diagram, separating a one-phase region from a two-phase region where the LDL and HDL patches coexist, separated by a first-order transition line^4,11,41−43^ (Supporting Information and Figure S1). The coexistence of these two structural motifs of water has been observed both *in silico*(41,44−46) and in experimental^21,47−53^ works, especially upon supercooling. Although further experimental evidence for inhomogeneous structures of liquid water and fluctuations between HDL and LDL patches was provided by the isosbestic point in the temperature-dependent OH stretching Raman signal,^54,55^ temperature-dependent infrared spectra of liquid water,^56^ optical Kerr effect measurements,^57^ and X-ray absorption and emission spectroscopy,^42,47−49,58^ the coexistence of these fluctuations under ambient conditions and their implications remain elusive and controversial.^14,16,21,59−66^ Nonetheless, showcasing two distinct arrangements of water molecules and their fluctuations is crucial, as it has the potential to revolutionize our understanding of biochemistry and reveal that life-supporting conditions may hinge on the presence of two kinds of H-bond organization in liquid water.^1,3,67^

Little is known about the topology of liquid water, specifically regarding the existence of motifs forming the H-bond networks such as rings, clathrates, and clusters, which, although numerically proposed through molecular dynamics,^45,68−70^ have not been experimentally observed. This knowledge gap arises because techniques commonly employed to investigate density fluctuations in liquid water and H-bond network structures are limited to a length scale of ∼1 nm (Figure 1). Therefore, there is a strong demand for experimental techniques for microscopically deciphering H-bond structures in liquid water as well as in aqueous solutions of electrolytes, suspensions of biomolecules, and inorganic materials. These systems have garnered more attention due to their potential to unveil the intricate relationship among charged interfaces, high- to low-density liquid fluctuations, and their role in the formation of large-scale H-bond supramolecular structures, as suggested by molecular dynamics simulations of the hydration shell of the lysozyme protein.^71^ Supramolecular structures of orientationally ordered water as large as 1 μm have been reported in light scattering measurements of aqueous solutions of low-molar mass compounds^72−74^ and in wide-field second harmonic (SH) microscopy of divalent cations interacting with water and negatively charged free-standing lipid bilayers,^75,76^ although not explicitly connected to HDL and LDL domains.

**Figure 1:**
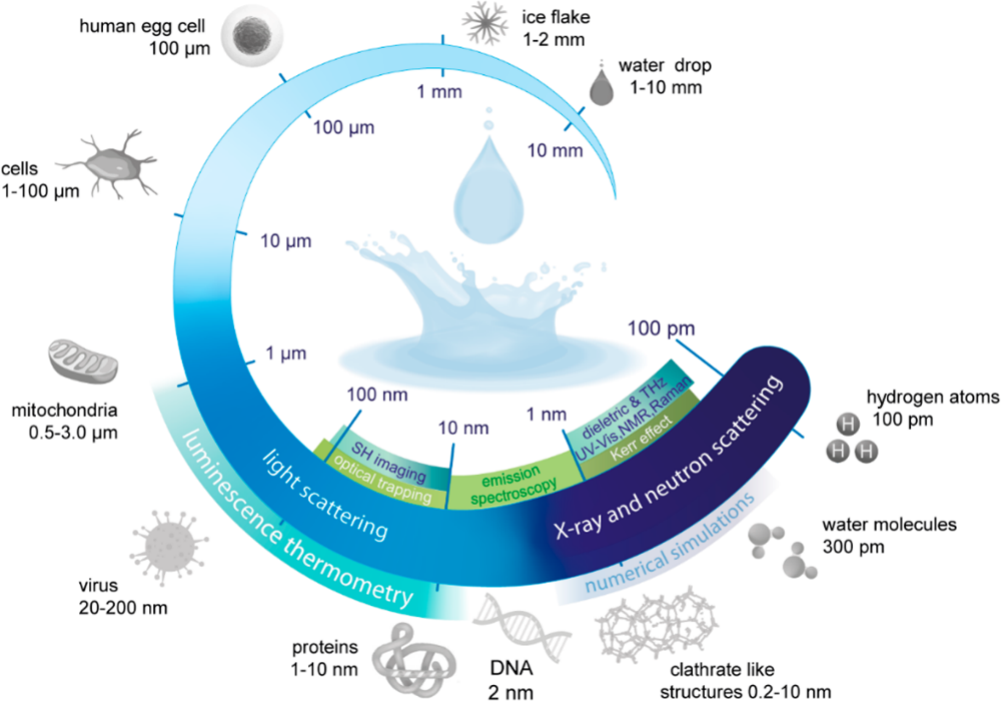
Infographic of the various techniques used to investigate anomalies in liquid water across different length scales. The temperature dependence of the H-bond networks has been explored at different length scales. While X-ray and neutron scattering, numerical simulations, and Kerr effect, dielectric, terahertz, ultraviolet–visible, nuclear magnetic resonance, and Raman spectroscopies operate at the length scale of hydrogen atoms and water molecules, SH imaging works at longer scales. Light scattering and luminescence nanothermometry, as shown in this work, can also be used up to a submicrometer length scale.

In recent years, there has been extensive research into the temperature dependence of the optical properties of a wide range of water-suspended materials such as quantum dots,^52^ plasmonic^77^ and luminescent^78^ nanoparticles, organic molecules,^79−81^ and trivalent lanthanide-based materials,^82−84^ including upconverting nanoparticles (UCNPs).^85−87^ The temperature (*T*) at which these materials exhibit a notable change in their optical properties is often termed the crossover temperature (*T*_c_).^52^ It predominantly falls within the range of 320–340 K and coincides with the minimum of the isothermal compressibility of water.^50^ Surprisingly, although some of these measurements have been interpreted in light of the two-state model of water,^52,80^ the observed bilinear trend has not been explicitly attributed to the presence of HDL and LDL motifs or the fluctuations between them. To the best of our knowledge, only one research paper has explored the intriguing relationship between the bilinear temperature dependence of the instantaneous Brownian velocity of NaYF_4_:Yb/Er UCNPs suspended in water and the high- to low-density liquid fluctuations.^87^ These experimental data, corroborated by molecular dynamics simulations, elucidated a geometric phase transition in which the LDL phase percolates below 330 K. *T*_c_, in this context, was interpreted as the onset of fluctuations between high- and low-density liquid water.^87^

In this work, we delve into the unique ability of upconversion nanothermometry^88^ to measure the temperature and pH dependence of the Brownian velocity^89^ of UCNPs of varying sizes (15–106 nm in diameter) dispersed in water [so-called nanofluids (section 2 of the Supporting Information)]. We estimate an upper-temperature threshold for the liquid water density fluctuations in the region dominated by HDL domains under ambient conditions, which agrees with the value suggested in the hypothetical phase diagram of liquid water under ambient conditions (Figure S1).^4,8,11,23,31,90^ We also show that increasing the pH of the nanofluids fragments the LDL domains (the LDL–HDL fluctuations become less favorable), similar to increasing the pressure in this phase diagram. Furthermore, our results provide new insights into the relative proportion of HDL and LDL motifs that coexist in the hydration water around the particle surface under ambient conditions. We find that the high- to low-density liquid fluctuations depend on the size of the suspended nanoparticle and the pH of the nanofluid. As the size increases, the relative proportion of HDL domains increases, while as the surface charge increases (controlled by pH), the relative proportion of LDL patches increases.

We measured the instantaneous Brownian velocity of 15 nm [diameter (*d*)] NaGdF_4_:Yb/Er(18%/2%)@NaGdF_4_ core–shell UCNPs dispersed in water (H_2_O, pH 5.10), heavy water (D_2_O), and ethanol (EtOH) at a volume fraction of 0.085% (Figure 2a and Table S1). The colloidal stability of the nanofluids and the size distribution of the UCNPs are presented in Figures S2–S6 and Table S2. The experimental setup, similar to that in ref (89), is described in sections 2 and 3 of the Supporting Information, Figures S7–S10, and Table S3. As the solvent density (ρ) increases, the Brownian velocity decreases because denser liquids have a higher effective mass (defined as the combined mass of the UCNPs and half of the liquid mass moving cooperatively with them).^91^ This is well illustrated by the difference in density between EtOH and D_2_O (781 and 1105 kg m^–3^, respectively, at 303 K^92^). The lower density of EtOH facilitates the faster motion of the UCNPs within the nanofluid, whereas the higher density of D_2_O results in considerably slower motion (Figure 2a). An analogous density dependence was observed in the Brownian velocity of UCNPs containing an oleic acid coating and dispersed in toluene and cyclohexane, where the increase in solvent density decelerates the motion of particles.^87^

**Figure 2:**
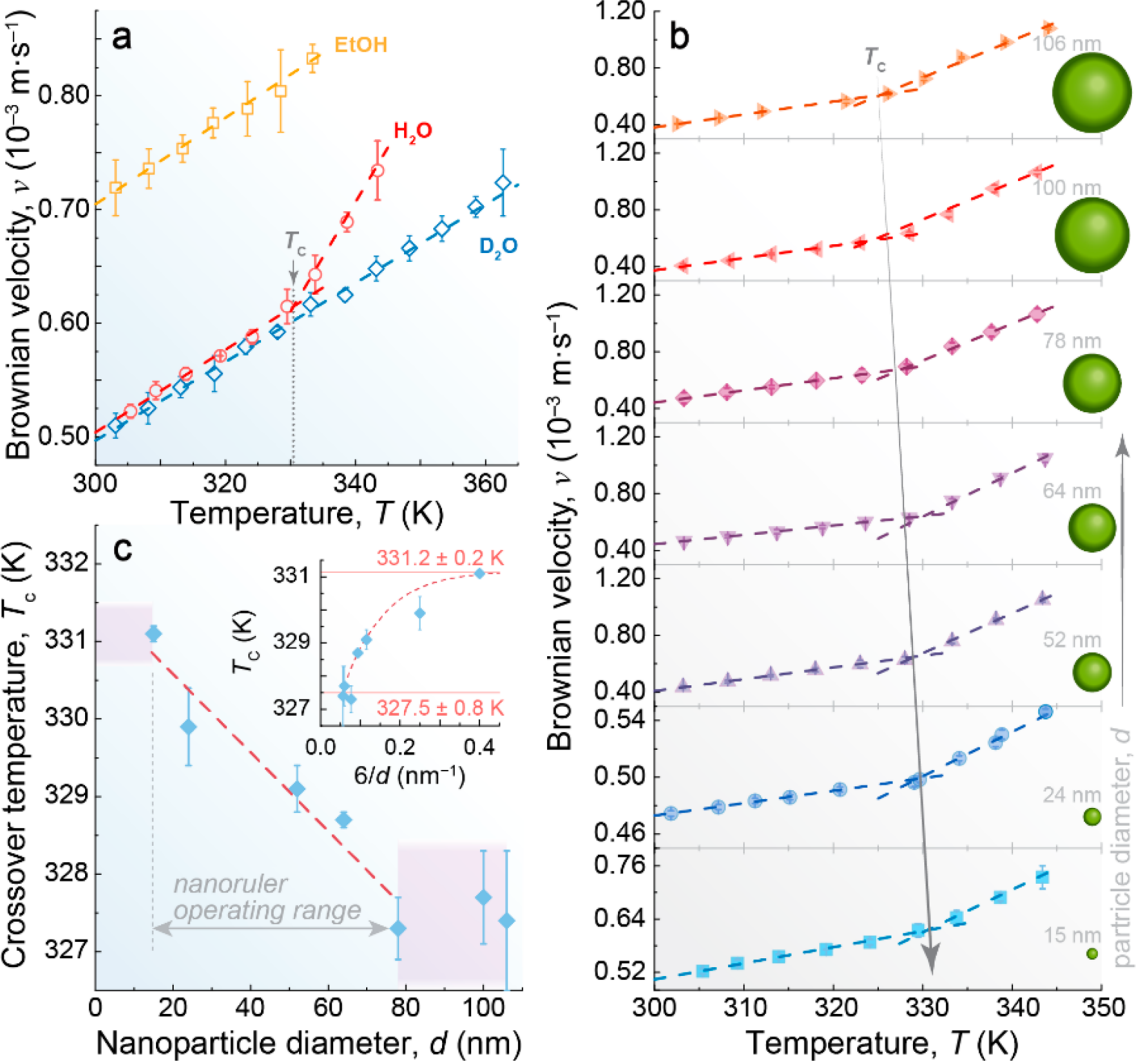
Solvent effect and size dependence in the Brownian velocity of UCNPs. (a) Temperature-dependent Brownian velocity of the 15 nm UCNPs suspended in EtOH, H_2_O, and D_2_O. The gray arrow highlights the existence of a crossover temperature in the water-suspended UCNPs around 330 K, indicating the anomalous behavior of water. (b) Temperature-dependent Brownian velocity of different-sized UCNPs (15–106 nm) at pH 5.10. (c) Size-dependent crossover temperature of the nanofluids from panel b, where the red dashed line is a guide for the eyes, highlighting the operating range of sizes that can be used to probe the different motifs of liquid water. The inset presents the dependence of *T*_c_ on the surface/volume ratio (*S*/*V* = 6/*d*). The lines are guides for the eyes.

The Brownian velocity of the UCNPs depends on the particle size, decreasing by ∼20% when the size increases from 15 to 64 nm and being almost independent of size for larger values of ≤106 nm, as observed in Figure 2b and Figure S11a. Due to the same volume fraction used across all colloidal suspensions (0.085%), smaller particle sizes correspond to a greater number of UCNPs in the suspensions (Figure S11 and Table S4). Consequently, this results in an increased Brownian velocity of the UCNPs with a higher particle count in the suspension. The number of UCNPs increases by a factor of ∼4 when the particle size decreases from 106 to 64 nm. However, this increment is far more striking, reaching a factor of 40, when the size decreases from 52 to 15 nm. The observed decrease in Brownian velocity as UCNP size increases is therefore attributed to the dependence of Brownian velocity on the number of particles per unit of volume in the suspensions due to the particle–particle interactions. It is noteworthy that these findings align seamlessly with our earlier observations.^89^

Notably, the Brownian velocity of the UCNPs in the aqueous nanofluids exhibits a bilinear trend, regardless of the size of the particles (Figure 2b). This behavior is attributed to the presence of two distinct motion regimes for the UCNPs. When *T* < *T*_c_ (300–330 K), there are HDL fluctuations into more voluminous LDL regions within the HDL dominant phase. Consequently, this gives rise to a greater effective mass of the nanoparticles, resulting in lower Brownian velocity values. Conversely, when the temperature exceeds the critical threshold (*T* > *T*_c_), density fluctuations cease because all LDL motifs have already been converted into HDL ones. This leads to a liquid state characterized by localized fluctuations within the HDL phase, leading to higher Brownian velocity values. It is worth stressing that the obtained *T*_c_ value closely corresponds with the minimum value of the isothermal compressibility of liquid water, which is related to the change from a more to a less organized tetrahedral organization due to the density increase.^50^ Once isothermal compressibility depends on fluctuations in density indicating a relative change in the volume, the similarity between the *T*_c_ values of the Brownian velocity of UCNPs and the minimum isothermal compressibility of liquid water is explained by the change in volume of the HDL and LDL motifs with an increase in temperature.^93^

Compared to liquid water, the weaker hydrogen bonds in ethanol^94^ result in a continuous linear increase in the Brownian velocity of UCNPs upon heating. Although the low boiling point of EtOH limits its study at temperatures above 333 K, the lack of tetrahedral arrangements means that *T*_c_ is not expected to occur.^95^ While HDL and LDL motifs have also been identified in liquid D_2_O,^96,97^ the presence of isotopic quantum effects generates a more ordered structure that enhances thermodynamic stability,^98,99^ displacing the Widom line toward higher temperatures (4 K at ∼0 bar).^31^ The melting point (4 K), maximum density (7 K), isothermal compressibility (5 K), nuclear quantum effects (5 K), and viscosity (−6.5 K) also exhibit a temperature offset in D_2_O relative to the values in H_2_O.^100−104^ Then, *T*_c_ might be shifted by a few degrees. No crossover temperature, however, was observed in the temperature dependence of the Brownian velocity of the UCNPs in D_2_O (Figure 2a).

Interestingly, recent findings on the three-dimensional confinement of light and heavy water within zwitterionic liposomes of different sizes, as determined through SH imaging and scattering experiments, have yielded a noteworthy conclusion: The H-bond networks in D_2_O differ not only at subnanometer length scales but also at length scales of ≲100 nm.^76^ This significantly exceeds the previously observed confinement length scales of ∼2–20 nm.^76^ On the contrary, experimental^105^ and simulation^106,107^ results have shown that the dielectric constant of confined water is much lower than that of bulk water. Similarly, Kim et al.^108^ highlighted how the confinement of interfacial water molecules caused by surface charge results in a lower dielectric constant at the hydration layer, which can be controlled by changing the temperature at the surface. Therefore, the Brownian velocity of UCNPs dispersed in heavy water displays an uninterrupted linear increase from room temperature to the boiling point, similar to the behavior observed for water at temperatures below *T*_c_ (Figure 2a), because of the much larger spatial extent over which H_2_O molecules interact in the hydration water of the UCNPs, corresponding to a much larger spatial extent for low- to high-density liquid water fluctuations.

We observed a pronounced reduction in the *T*_c_ values of the nanofluids as the diameter *d* of the UCNPs increased (Figure 2c). This intriguing size-dependent trend shows that, as *d* → 0, *T*_c_ converges toward 331.2 ± 0.2 K, as nicely illustrated by the surface/volume (*S*/*V*) ratio of the UCNPs [quasi-spherical morphology (Figure S5)] in the inset of Figure 2c. This temperature should therefore correspond to the onset temperature of the fluctuations between high- and low-density liquid states in pure water (*d* = 0 in Figure 2c). Interestingly, this upper-temperature threshold for fluctuations in the HDL domain-dominated region under ambient conditions agrees with the value (325.0 ± 1.0 K) estimated based on data from the hypothetical phase diagram of water under ambient conditions published in refs (4) and (8) (Figure 3).

**Figure 3:**
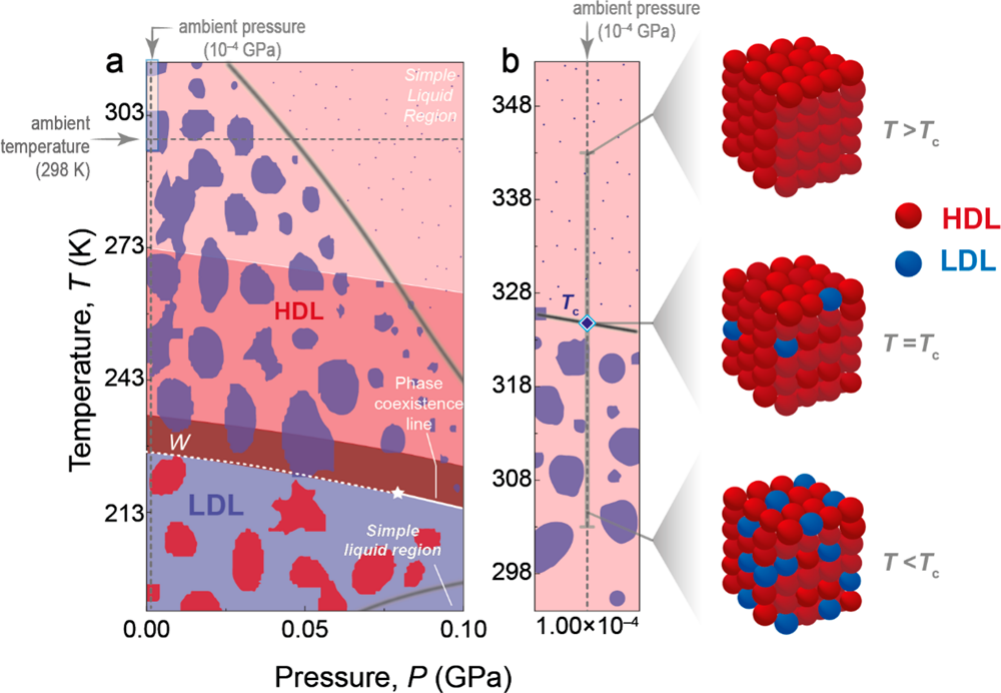
Hypothetical phase diagram of liquid water. (a) Coexistence of HDL (red) and LDL (blue) domains near the Widom line (W). Below W, LDL dominates with fluctuations in HDL domains, whereas above W, HDL dominates with LDL fluctuations. The white star represents the liquid–liquid critical point. With greater distance from the critical point, fluctuations decrease in size, as indicated by the blobs. The gray line outlines the “funnel of life”, where water exhibits unusual properties crucial for maintaining life. Outside the funnel, at higher temperatures, only local fluctuations occur in the HDL liquid (indicated by small blue dots on the red background). Reproduced with permission from ref (8). Copyright 2019 Springer Nature. (b) Close-up of the shaded area in panel a showing the upper-temperature limit of the “funnel of life” at ambient pressure, corresponding to crossover temperature *T*_c_ (diamond), and illustrative schemes of the temperature dependence of high- to low-density liquid fluctuations.

Furthermore, we hypothesize that the decrease in the *T*_c_ of the aqueous nanofluids derived from Figure 2c compared to pure water stems from a decrease in the high- to low-density liquid water fluctuations as a consequence of the prevalence of a higher concentration of HDL patches relative to LDL regions in the volume of nanofluid moving cooperatively with UCNPs. Notably, this hypothesis corroborates previous findings, both *in silico*(71) and in experiments,^109^ about the hydration water of the lysozyme protein. This hydration water (defined as the water molecules encompassing the protein within a 0.6 nm shell)^71^ exhibits local distortions when compared to bulk water. For instance, its density is much higher than that of the bulk^110^ and the dielectric constant of interfacial water in the double layer is much lower than that of bulk water.^108^ These distortions induce a different ordering of water molecules at the interface, characterized by a higher concentration of HDL domains than LDL domains, compared to bulk water.^71,109^ Like the impact of the lysozyme protein in liquid water, the presence of UCNPs also influences the local structure of water within the aqueous nanofluids. Consequently, nanofluids with larger UCNPs (lower *S*/*V* ratios) contain a relatively higher proportion of HDL domains in the hydration shell of the nanoparticles, leading to lower *T*_c_ values (Figure 2c). When *d* > 78 nm, *T*_c_ reaches a plateau at ∼327.5 K. This occurs because the *S*/*V* ratio changes negligibly beyond this size threshold. Remarkably, the *T*_c_ of ligand-free and silica-coated UCNPs of comparable size is similar (Figure 2c), suggesting an analogous relative HDL/LDL proportion at the particle surface, in agreement with an identical charge density of the water–silica and water NaYF_4_ interfaces [|ζ| ∼ 35 mV (Figures S3 and S4)].

The vicinity of the UCNPs can be sensitively influenced by local ions and ligands, with effects already occurring at extremely low concentrations.^111^ Fine-tuning the pH of suspensions at the water–silica interface was found to induce changes in charge density, impacting the orientation of water molecules.^110,112^ Recent surface-enhanced IR absorption spectroscopy results have also shed light on the influence of pH on hydrogen and water binding energies on platinum surfaces.^113^ Also, a pH dependence of the onset temperature of the anomaly related to the minimum isothermal compressibility of liquid water was reported for aqueous suspensions, including 1-methyl-5-nitroindoline,^80^ Eu^3+^ complexes,^82,83^ and NaYF_4_:Yb/Er UCNPs.^86^ These findings suggest a potential role of pH in influencing high- to low-density fluctuations within aqueous nanofluids.

To explore this possibility, we evaluated the temperature-dependent Brownian velocity of the UCNPs dispersed in aqueous nanofluids while systematically varying the pH values from 2.70 to 8.50 (Figure 4a for *d* values of 15, 64, and 78 nm and Figure S12 for *d* values of 24, 52, and 106 nm). Except for pH values between 7.0 and 8.0, the measured absolute ζ potential values (|ζ| > 20 mV) indicate stability with no UCNP aggregation (Figures S3 and S4).

**Figure 4:**
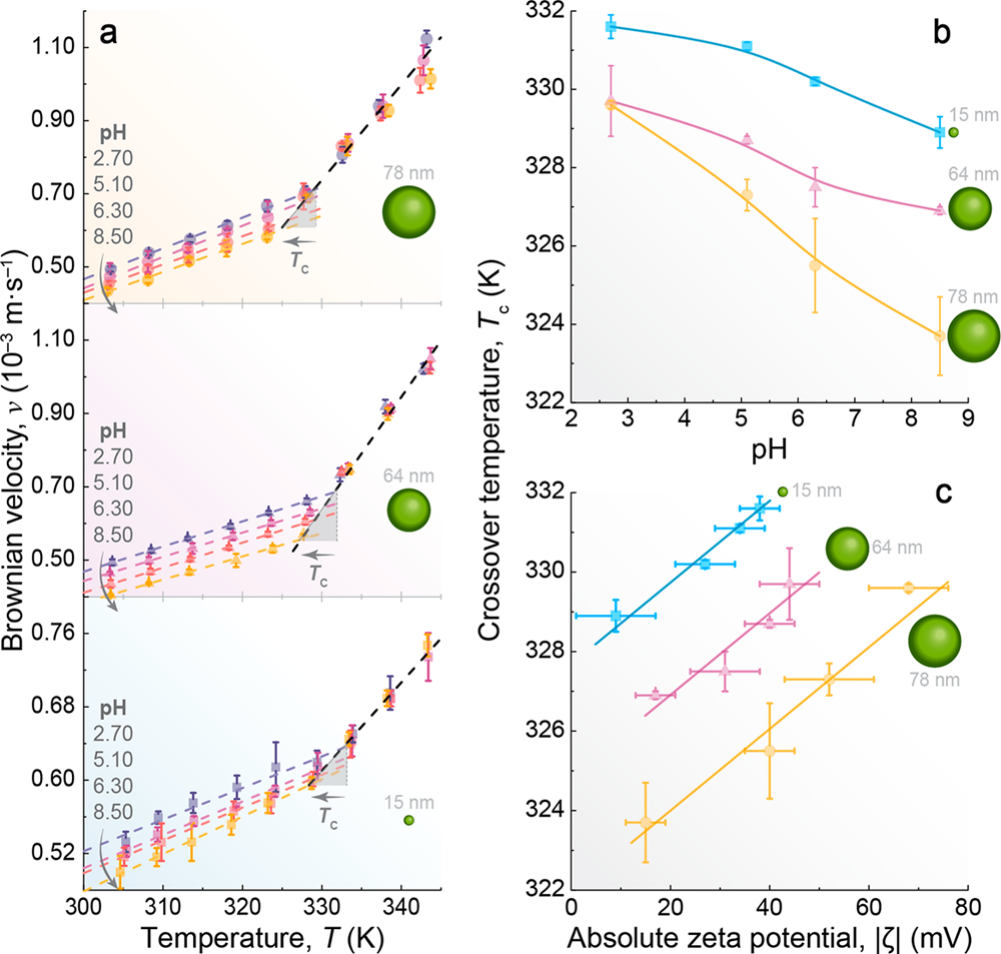
Correlation among the crossover temperature, pH, and ζ potential. (a) Effect of pH on the Brownian velocity of UCNPs with diameters within the operating range of the nanorulers, as defined in Figure 2c. The dashed lines are the best linear fits at each pH for *T* < *T*_c_ and the same linear fit for all of the pH values for *T* > *T*_c_ (*r*^2^ > 0.98 for all samples). *T*_c_ as a function of (b) pH and (c) |ζ|. The solid lines are guides to the eyes.

pH exerts a notable influence on Brownian velocity, with an increase in pH leading to a reduction in Brownian velocity when *T* < *T*_c_, while the pH dependence becomes negligible when *T* > *T*_c_. This impact is primarily on LDL domains, present only when *T* < *T*_c_. Increasing the pH weakens the H-bond network due to an increased concentration of OH^–^ ions, disrupting the tetrahedral organization of LDL domains due to their voluminous planar structure.^114^ Increasing the pH triggers the fragmentation of LDL domains into smaller ones,^8^ while keeping the HDL/LDL ratio, leading to a deceleration of the motion of the UCNPs. This effect mirrors the application of high pressures, as suggested by previous neutron diffraction measurements^114,115^ and Monte Carlo simulations.^116^ As the pressure increases at a constant temperature, the LDL–HDL fluctuations become less favorable.^8^ This can be visualized as the fragmentation of the LDL domains (Figure 3 and Figure S1). However, recent machine learning-based molecular dynamics simulations of ions in salt water have shown that the ions do not homogeneously distort the structure of water but instead have localized structural effects in the first solvation shell.^117^ Our results agree with this scenario as increasing the pH of the nanofluids is an ingenious strategy for simulating a pressure-like effect and evaluating microscopic changes in H-bond networks.

The decrease in Brownian velocity with an increase in pH results in a concomitant decrease in *T*_c_ values (Figure 4b), indicating that low- to high-density liquid fluctuations at nanoparticle surfaces cease at lower temperatures in basic media compared to acidic media. To explain this dependence, we consider the influence of pH on the surface charge of UCNPs, with experiments demonstrating how surface charge controls the water structure near the interface. The fine-tuning of pH affects the extent of orientation of water molecules near the interface, as reported for water–silica interfaces.^108,110^ Therefore, to study the effect of pH on the electric double layer of UCNPs, we measured the ζ potential of the distinct aqueous nanofluids. Our results show a decrease in |ζ| as pH increases (Figure S4), in good accordance with previous reports on upconverting^118^ and plasmonic^111^ nanoparticles. This mirrors the trend observed for *T*_c_ (Figure 4b and Figure S13), highlighting how the presence of ions in the medium can affect the surface charge of the UCNPs. The increase in the extent of electrostatic repulsion of the UCNPs causes a higher *T*_c_ value (Figure 4c). As higher *T*_c_ values correspond to larger amounts of LDL patches, as discussed above, we propose that a greater surface charge (or the electrical potential at the slipping plane) increases the proportion of LDL patches in the hydration water of the nanoparticles. This hypothesis agrees with simulations conducted by Gallo’s group, indicating a slower decrease in the rate of LDL domains at the biomolecule interface with an increase in temperature.^71^ Moreover, for a constant |ζ| value, nanofluids with smaller UCNPs exhibit a higher *T*_c_, consistent with the findings depicted in Figure 2c. It is noteworthy that the same conclusion can be drawn exclusively from upconversion thermometry and by combining upconversion thermometry with ζ potential measurements. Similar results were reported by Kim et al.,^108^ demonstrating how temperature changes the interfacial structure of water by mitigating the effect of surface charge at water–oil interfaces.

Interestingly, Barisik et al.^119^ showed that, at a constant pH, the surface charge density of Si-NPs decreases with an increase in particle size until it stabilizes after reaching a critical diameter of 100 nm. A similar trend was observed for metal oxide nanoparticles.^120^ These findings align with our *T*_c_ size dependence results (Figure 2c), revealing a decrease in *T*_c_ with an increase in particle size up to a critical value (>78 nm), beyond which it remains constant.

In summary, our study systematically investigated the impact of temperature and pH on Brownian velocity in a range of UCNPs (15–106 nm diameter) dispersed in water. We consistently observed a decrease in the onset temperature of high- to low-density liquid water fluctuations with an increase in nanoparticle size, indicative of an increased presence of HDL domains at nanoparticle surfaces. UCNPs, therefore, behave as nanorulers for assessing the HDL/LDL proportion in surrounding hydration water. Moreover, the upper-temperature threshold for these fluctuations, as predicted by our experiments, agrees with values proposed in the hypothetical phase diagram of water under ambient conditions based on the two-state model. Additionally, we have shown that increasing the pH decreases *T*_c_ and decreasing the *T*_c_ decreases the relative amount of LDL patches, akin to external pressure on pure water. By precisely controlling the UCNP size and pH levels, we have simulated the effects of temperature and pressure on HDL and LDL hydrogen bonding networks, mirroring predictions in the hypothesized phase diagram. Within nanofluids, the local environment around nanoparticles exerts a significant influence on their physical–chemical properties, being different from the bulk due to interaction with the particle surface.^111^ This work elegantly underscores the substantial impact of these interactions, serving as compelling evidence of this effect for a specific example of luminescent nanoparticles.

These findings resonate with the intricate interplay between water and various nonpolar media, metals, oxides, and biomembranes, emphasizing the importance of the water charge-asymmetrical molecular configuration at interfaces.^75,108,110^ In conclusion, our results provide compelling experimental evidence regarding the significance of the size of suspended nanoparticles or biomolecules in understanding the dynamics of high- to low-density liquid fluctuations and water behavior at charged interfaces.

## Methods

*Synthesis of Upconverting Nanoparticles.* NaGdF_4_:Yb/Er(18/2%)@NaGdF_4_ (average diameter of 15 nm, core–shell), NaYF_4_:Yb/Er(18/2%)@NaYF_4_ (average diameter of 24 nm, core–shell), NaYF_4_:Lu/Yb/Er(40/18/2%) (average diameter of 52 nm, core-only), NaYF_4_:Lu/Yb/Er(47/18/2%) (average diameter of 64 nm, core-only), NaYF_4_:Lu/Yb/Er(47/18/2%) (average diameter of 78 nm, core-only), NaYF_4_:Lu/Yb/Er(47/18/2%)@SiO_2_ (average diameter of 100 nm, core–shell), and NaYF_4_:Lu/Yb/Er(50/18/2%) (average diameter of 106 nm, core-only) ligand-free UCNPs were synthesized through a coprecipitation method based on a previous report.^121^ The detailed synthesis procedure and the characterization of the UCNPs are described in section 2 of the Supporting Information.

*Preparation of the Nanofluids.* Aqueous nanofluids containing ligand-free UCNPs were obtained by adjusting the pH of water between 2.70 and 5.10 by adding aqueous solutions of sodium hydroxide and hydrochloric acid (0.1 mol L^–1^) at a volume fraction (ϕ) of 0.085%. The aqueous nanofluids of 15 nm UCNPs were freeze-dried, and the resulting powder was dispersed in heavy water and ethanol under sonication to obtain the corresponding nanofluids at ϕ = 0.085%. A detailed description of pH measurements and the preparation of nanofluids is presented in section 2 of the Supporting Information.

*Upconversion Spectroscopy.* The upconverting emission spectra of the nanofluids were recorded using the experimental setup shown in Figure S7. A quartz cuvette (9F-Q-10, Starna Cells) filled with 0.50 mL of the nanofluids was irradiated with a near-infrared 980 nm laser diode (DL980-3W0-T, CrystaLaser) operating with a power density (*P*_D_) of 62 W cm^–2^. The excitation radiation was collimated with a plano-convex lens (LA1145-AB, Thorlabs). The upconversion emission spectra were registered with a USB portable spectrometer (Maya 2000 Pro, Ocean Insight) coupled to an optical fiber (P600-1-UV-vis, Ocean Insight). A short pass filter (FESH0750, Thorlabs) was used to cut off the 980 nm laser signal from the emission spectra. The temperature of the nanofluids was increased through the Joule effect by attaching a Kapton thermofoil heater (HK6906, Minco) in thermal contact with one side of the cuvette containing the nanofluids. This setup allows us to control both the initial temperature and the temperature increase. Further information is provided in section 2 of the Supporting Information.

*Measurement of Temperature through Upconversion Nanothermometry.* The luminescence intensity ratio between the emission bands corresponding to the Er^3+^^2^H_11/2_ → ^4^I_15/2_ (*I*_H_, 510–534 nm) and ^4^S_3/2_ → ^4^I_15/2_ (*I*_S_, 534–554 nm) transitions was used to define a thermometric parameter (Δ = *I*_H_/*I*_S_) and predict absolute temperature *T* of the nanofluids as detailed in section 3 of the Supporting Information.

*Determination of the Brownian Velocity of the UCNPs in the Nanofluids.* The emission spectra were recorded at different distances *x*_*i*_ from the Kapton thermofoil heater to construct time-dependent temperature profiles through upconversion nanothermometry. An excellent linear correlation between *x_i_* and the onset time (the time at which the temperature increases above its uncertainty) was systematically obtained. The slope of the linear fit to each data set represents the Brownian velocity of the UCNPs in the nanofluids. Further details are provided in sections 4–6 of the Supporting Information.

## Supporting Information Available

Description of the two-state model and a hypothetical phase diagram of liquid water, nanoparticle synthesis, characterization of materials, and a study of the thermometric performance and an investigation of the instantaneous Brownian velocity of upconverting nanoparticles (PDF)
Transparent Peer Review report available (PDF)
