# Supplementary material for: Deciphering Density Fluctuations in the Hydration Water of Brownian Nanoparticles via Upconversion Thermometry
Source: J Phys Chem Lett. 2024 Feb 29;15(9):2606–15. doi: 10.1021/acs.jpclett.4c00044 (PMC10926164; doi:10.1021/acs.jpclett.4c00044)
Supplement: Supplementary file 1 — jz4c00044_si_001.pdf [file jz4c00044_si_001.pdf]

Supporting Information

Deciphering Density Fluctuations in the Hydration Water of Brownian Nanoparticles via Upconversion Thermometry

Fernando E. Maturi[1,2], Ramon S. Raposo Filho[1], Carlos D. S. Brites[1], Jingyue Fan[3], Ruihua He[3],

Bilin Zhuang[4], Xiaogang Liu[3], and Luís D. Carlos[1]**

[1]Phantom-g, CICECO - Aveiro Institute of Materials, Department of Physics, University of Aveiro, 3810-193 Aveiro, Portugal.

[2]Institute of Chemistry, São Paulo State University (UNESP), 14800-060 Araraquara-SP, Brazil.

[3]Department of Chemistry, National University of Singapore, 117543 Singapore, Singapore.

[4]Harvey Mudd College, 301 Platt Boulevard, California 91711, USA.

Corresponding Authors

*Xiaogang Liu, E-mail: chmlx@nus.edu.sg

*Luís D. Carlos, E-mail: lcarlos@ua.pt

This PDF file includes:

Materials and Methods
Supplementary Text
Supplementary Figures S1 to S13
Supplementary Tables S1 to S4
References

Table of Contents

1. The two-state model and a hypothetical phase diagram of liquid water S3

2. Synthesis and characterization S4

3. Experimental setup for temperature-dependent photoluminescence measurements.................S7

4. Temperature mapping through upconversion nanothermometry S7

5. Data denoising and determination of the instantaneous ballistic Brownian velocity................S9

6. Calculation of crossover temperature...S10

7. Equipartition theorem...S10

8. Brownian velocity and number of UCNPs per mL in the nanofluids S11

9. Supplementary Figures...S12

10. Supplementary Tables S25

11. References S27

1. The two-state model and a hypothetical phase diagram of liquid water

The strongest evidence supporting the two-state model of water comes from the liquid-liquid phase transition (LLPT) hypothesis proposed by Poole, Sciortino, Essmann, and Stanley based on molecular dynamics simulations.[1] According to this hypothesis, a second critical point for water in the supercooled regime separates the LDL from the HDL in a discontinuous phase transition.[2-3] At the molecular level, the spacious LDL is formed when water molecules in the first hydration shell assemble in a more organized tetrahedral hydrogen-bonding network, while the more tightly connected HDL forms when an additional water molecule from the second hydration shell enters the first hydration shell, disrupting the LDL organization and creating a smaller and distorted hydrogen-bonding motif.[4] Direct observations of the LLPT are difficult due to the quick crystallization of supercooled liquid water, which only exists in one state below 215 K.[5-6] Nevertheless, recent studies of isothermal volume changes in diluted polyol and trehalose aqueous solutions under varying pressures have confirmed the existence of two states of water experiencing an LLPT between the metastable LDL and HDL,[7-8] corroborating the hypothesis that pure water undergoes an LLPT as well. Furthermore, the conversion between HDL and LDL has been experimentally observed by Kim *et al.*.[9]

The LLPT hypothesis offers a fresh perspective for understanding the singular behavior of liquid water in terms of LDL and HDL motifs at varying pressures and temperatures, as demonstrated by the hypothetical phase diagram depicted in **Figure S1**.[10] The phase diagram portrays the liquid-liquid coexistence line between LDL and HDL in terms of simple liquid regions. Additionally, the diagram includes the liquid-liquid critical point (LLCP), which may be either real or virtual, the Widom line (W) marking the crossover between the metastable and stable regions in the one-phase region, and fluctuations on various length scales emerging from LLCP, resulting in local spatially separated regions in the anomalous region. The amorphous solid states of LDL and HDL can exist at extremely low temperatures as low-density (LDA, low pressure) and high-density (HDA, high pressure) amorphous ice, respectively. In fact, recent findings have also demonstrated the possibility of obtaining medium-density amorphous ice under specific conditions of pressure and temperature.[11] This discovery indicates that the proposed phase diagram still has room for optimization and potential for further improvements.

2. Synthesis and characterization

Materials. Yttrium(III) acetate hydrate (99.9%), ytterbium(III) acetate hydrate (99.9%), erbium(III) acetate hydrate (99.9%), lutetium(III) acetate hydrate (99.9%), ammonium fluoride (>98%), 1-octadecene (90%), oleic acid (90%), cyclohexane (99.5%), absolute ethanol (>98%), methanol (99.9%), hydrochloric acid (37%), sodium hydroxide (>98%), deuterium oxide (99.9 atom % D), polyvinylpyrrolidone, and tetraethyl orthosilicate were purchased from Sigma-Aldrich and used as received.

Synthesis of $NaGdF_4$:Yb/Er(18/2%)@$NaGdF_4$ core-shell nanoparticles (15 nm). The synthesis of upconverting nanoparticles (UCNPs) was carried out according to a previously reported procedure.[12] In a 50 mL flask, an aqueous solution (2 mL) containing $Gd(CH_3CO_2)_3$ (0.097 g, 0.320 mmol), $Yb(CH_3CO_2)_3$ (0.030 g, 0.072 mmol), and $Er(CH_3CO_2)_3$ (0.003 g, 0.008 mmol) was mixed with oleic acid (4 mL) and 1-octadecene (6 mL) to obtain the core of UCNPs. The resulting mixture was heated at 423 K for 1.3 h, forming lanthanide oleate complexes, before cooling to room temperature. Subsequently, ammonium fluoride (1.2 mmol) and sodium hydroxide (1.0 mmol) dissolved in 5 mL of methanol were added and stirred at 318 K for 3.5 h. To remove the methanol, the reaction temperature was increased to 378 K. The solution was then heated to 553 K under an argon flow for 1.5 h, followed by cooling to room temperature. The resulting nanoparticles were precipitated by adding ethanol, collected by centrifugation, washed with ethanol, and finally redispersed in 4 mL of cyclohexane.

For the optically inert shell of the 15 nm UCNPs, an aqueous solution (2 mL) containing $Gd(CH_3CO_2)_3$ (0.121 g, 0.400 mmol) was combined with oleic acid (3 mL) and 1-octadecene (7 mL) in a 50 mL flask. The resulting mixture was heated at 423 K for 1 h under stirring and then cooled to 323 K. The as-synthesized $NaGdF_4$:Yb/Er(18/2%) 15 nm core nanoparticles, dispersed in 4 mL of cyclohexane, were then added to the flask, followed by the addition of a 6 mL methanol solution of ammonium fluoride (1.6 mmol) and sodium hydroxide (1.0 mmol). The mixture was stirred at 323 K for 30 min and then the reaction temperature was increased to 373 K. After removing the methanol, the solution was heated at 563 K under an argon atmosphere for 1.5 h and then cooled to room temperature. The resulting core-shell nanoparticles were precipitated by the addition of ethanol, collected via centrifugation, washed with ethanol, and redispersed in cyclohexane.

Synthesis of $NaYF_4$:Yb/Er(18/2%)@$NaYF_4$ core-shell nanoparticles (24 nm). The synthesis of 24 nm core-shell UCNPs followed the same procedure as described in the synthesis of the 15 nm core-shell UCNPs, except for replacing $Gd(CH_3CO_2)_3$ with $Y(CH_3CO_2)_3$.

Synthesis of $NaYF_4$:Lu/Yb/Er core-only nanoparticles (52, 64, 78, and 106 nm). In a typical experimental procedure, a 2 mL aqueous solution of $Ln(CH_3CO_2)_3$ (0.2 mol·L^{-1}, Ln = Lu, Y, Yb, and Er) was added to a 50 mL flask containing oleic acid (3 mL) and 1-octadecene (7 mL). The mixture was heated to 423 K for 1 h. After cooling to 323 K, a methanol solution (6 mL) containing ammonium fluoride (1.6 mmol) and sodium hydroxide (1.0 mmol) was added under stirring for 30 min. After removing the methanol through evaporation, the solution was heated at 563 K under argon for 3 h and then cooled down to room temperature. The resulting nanoparticles were washed several times with ethanol and redispersed in 4 mL of cyclohexane. The same procedure was used to obtain UCNPs of varying sizes. The heating duration, temperature, and concentration composition were tuned to achieve different doping ratios of Lu/Yb/Er: 40/18/2% (52 nm), 47/18/2% (64 and 78 nm), and 50/18/2% (106 nm).

Preparation of ligand-free nanoparticles. The as-synthesized oleate-capped UCNPs were dispersed in a solution containing 1 mL of ethanol and 1 mL of hydrochloric acid (2 mol·L^{-1}), followed by ultrasonication for 10 min to remove the oleate capping. The resulting ligand-free UCNPs were collected by centrifugation at 16,500 rpm for 20 min, washed with ethanol and deionized water several times, and then redispersed in deionized water. The same procedure was used for all the different-sized UCNPs, resulting in aqueous dispersions of ~100 mg·mL^{-1}.

Synthesis of $NaYF_4$:Lu/Yb/Er(47/18/2%)@SiO_2 core-shell nanoparticles (100 nm). In a standard method, an amorphous silica (SiO_2) shell was grown onto the surface of the 78 nm ligand-free UCNPs using polyvinylpyrrolidone (PVP) as a mediator.[13] PVP (250 mg) was dissolved under stirring in a 50 mL round-bottom flask containing 5 mL of deionized water and 15 mL of ethanol until its total dissolution. The ligand-free 78 nm $NaYF_4$:Lu/Yb/Er(47/18/2%) UCNPs (0.2f PVP mmol) were added to the flask and kept under stirring for 30 min. Then, a solution of tetraethyl orthosilicate (50 μL) dissolved in ethanol (300 μL) was added to the flask. The mixture was reacted overnight under stirring. Core-shell $NaYF_4$:Lu/Yb/Er(47/18/2%)@SiO_2 UCNPs were

collected via centrifugation at 16,500 rpm for 30 min and washed twice with ethanol. The silica-coated UCNPs were obtained with an average outer diameter of 100 nm (the thickness of the silica shell is 11 nm).

Preparation of the nanofluids. The pH of the solutions and suspensions was measured with a compact pH meter (SC S210-K, Mettler Toledo) at 293 K. The pH meter was calibrated by using a two-point calibration method with technical buffer calibration solutions at pH values of 4.01 and 10.01 (Mettler Toledo). Stock solutions with pH values of 2.70 ± 0.01, 5.10 ± 0.01, 6.30 ± 0.01, and 8.50 ± 0.01 were obtained by adding aqueous solutions of hydrochloric acid (0.1 $mol \cdot L^{-1}$) or sodium hydroxide (0.1 $mol \cdot L^{-1}$) to deionized water. Then, aqueous nanofluids were obtained at different pH values by dispersing the different-sized ligand-free UCNPs in the corresponding pH stock solutions under sonication. All nanofluids were prepared with a volume fraction (ϕ) of UCNPs of 0.085%. This choice ensures a sufficient signal-to-noise ratio in the photoluminescence studies while keeping the concentration of the UCNPs as low as possible, thus decreasing particle-particle interactions, known to increase Brownian velocity.[14] For the nanofluids obtained in heavy water (D_2O) and ethanol (EtOH), aqueous dispersions of 15 nm ligand-free UCNPs were freeze-dried to remove water by sublimation. The mass of dried UCNPs required to obtain $\phi = 0.085\%$ was then dispersed in the proper volume of D_2O or EtOH under sonication. **Table S1** summarizes the mass concentration (ρ_c) and density (ρ_n) according to the chemical composition of UCNPs used to obtain the different nanofluids at $\phi = \rho_c/\rho_n = 0.085\%$.

Colloidal characterization. The hydrodynamic diameter (d_H) of UCNPs was measured by dynamic light scattering (DLS) in a Malvern Zetasizer Nano ZS instrument (red badge ZEN3600, Malvern Instruments) operating with a 632.8 nm laser. To analyze the colloidal stability of UCNPs, zeta potential (ζ) measurements were carried out with the same instrument applying the Smoluchowski model. The d_H and ζ measurements were performed at 298 K in a folded capillary cell (DTS1070, Malvern Instruments). Three measurements with ten scans each were performed and the average values were used for data analysis. The resulting distribution of sizes and zeta potentials was adjusted to lognormal and Gaussian functions, respectively, using the mean and standard deviations to determine the average d_H and ζ and their uncertainties (**Figures S2–S4**, and **Table S2**).

Electron microscopy. Transmission electron microscopy (TEM) images of the UCNPs (**Figure S5**) were obtained using a field-emission transmission electron microscope (JEM-2100F, JEOL) operated at an acceleration voltage of 200 kV. The values of the diameters (*d*) and their corresponding uncertainties were retrieved from the mean and standard deviations of the lognormal function adjusted to the size distribution of the UCNPs (**Figure S6** and Table S2).

3. Experimental setup for temperature-dependent photoluminescence measurements

The emission spectra of UCNPs were recorded in the experimental setup presented in **Figure S7** (adapted from Reference [15]). The nanofluids were excited with a continuous-wave (CW) near-infrared laser diode (DL980-3W0-T, CrystaLaser) at 980 nm. The laser beam was collimated by a plano-convex lens (LA1145-AB, Thorlabs), resulting in a power density of 62 $W \cdot cm^{-2}$. The laser beam irradiates a semi-micro rectangular quartz cuvette (9F-Q-10, Starna Cells) filled with 0.50 mL of the nanofluids. The scanning position of the cuvette along the *x*-axis was controlled by a moving stage with a minimum step of 0.1 mm. Detection of the upconverting emission was performed by a USB portable spectrometer (Maya 2000 Pro, Ocean Insight) coupled to an optical fiber (P600-1-UV-VIS, Ocean Insight) using a short-pass filter (FESH0750, Thorlabs) to cut off the peak of the laser during spectral acquisition. Spectral acquisition was performed with a constant boxcar width (one pixel, 0.5 nm) and integration time of 250 ms. The temperature of the nanofluids was increased at one side of the cuvette through thermal contact by attaching it to a Kapton thermofoil heater (HK6906, Minco) mounted in a copper plate (4.6 × 2.5 cm^2) and coupled to a temperature controller (E5CN, Omron). The temperature controller is equipped with a K-type thermocouple (KA01-3, TME Thermometers) with a thermal resolution of 0.1 K.

4. Temperature mapping through upconversion nanothermometry

In the experimental setup depicted in Figure S7, the nanofluids were irradiated with a CW 980 nm laser diode until the stabilization of the temperature. After reaching different initial equilibrium temperatures (ranging from 303 to 343 K), one side of the cuvette (containing the nanofluids) was heated (temperature increment of 15 K for 300 s) and time-dependent upconversion emission spectra were recorded at different fixed positions along the *xx*-direction perpendicular to the laser beam (x_i = 0.0–6.0 mm, i = 1–4). For each time instant, the luminescence intensity ratio between the emission bands corresponding to $^2H_{11/2} \rightarrow ^4I_{15/2}$ (I_H, 510–534 nm) and $^4S_{3/2} \rightarrow ^4I_{15/2}$ (I_S, 534–554

nm) transitions was used to define the thermometric parameter ($\Delta = I_H/I_S$) and calculated the absolute temperature (T) as:[16]

$$\frac{1}{T} = \frac{1}{T_0} - \frac{k_B}{\Delta E}\ln\left(\frac{\Delta}{\Delta_0}\right) \quad \textbf{(S1)}$$

where ΔE is the energy separation between the Er^{3+} $^2H_{11/2}$ and $^4S_{3/2}$ thermally coupled levels, k_B is the Boltzmann constant, and Δ_0 is the thermometric parameter at room temperature (T_0). The value of ΔE was calculated as the difference between the barycenters of the $^2H_{11/2}$ (~525 nm) and $^4S_{3/2}$ (~545 nm) emitting levels (**Figure S8a**). Since Δ_0 corresponds to the value of Δ without laser-induced heating, its value can be obtained from the intercept of the curve of Δ measured as a function of the laser power density (Figure S8b). The thermometric parameter Δ increases as the temperature rises because the relative population between the $^2H_{11/2}$ and $^4S_{3/2}$ levels is in thermal equilibrium, following Boltzmann's distribution.[16] Therefore, this approach provides a reliable parameter to record time-dependent temperature profiles based on the emission spectra of the nanofluids containing UCNPs while heating them (Figure S8c).

The thermal sensing ability of the nanofluids can be assessed by the relative thermal sensitivity (S_r) and uncertainty in temperature (δT), which are the two figures of merit commonly used to compare the thermometric performance of luminescent thermometers. The value of S_r represents the Δ change per degree of temperature (in %·K^{-1}):

$$S_r = \left|\frac{1}{\Delta}\frac{d\Delta}{dT}\right| = \frac{\Delta E}{k_B T^2} \quad \textbf{(S2)}$$

where $d\Delta/dT$ is the rate of change of Δ in response to the variation of temperature, k_B is Boltzmann's constant, and T is the absolute temperature. The value of δT corresponds to the smallest temperature resolvable by the thermometer (in K):

$$\delta T = \frac{1}{S_r}\frac{\delta\Delta}{\Delta} \quad \textbf{(S3)}$$

where $\delta\Delta/\Delta$ is the relative uncertainty of Δ. The maximum relative thermal sensitivity and minimum uncertainty at the temperature obtained for each nanofluid are summarized in **Table S3**.

5. Data denoising and determination of the instantaneous ballistic Brownian velocity

The absolute temperature values measured by upconversion nanothermometry were computed as the reduced temperature θ(t):

$$\theta(t) = \frac{T(t) - T_i}{T_f - T_i} \qquad \textbf{(S4)}$$

where $T(t)$, T_i, and T_f are the instantaneous, initial, and final values of the temperature, respectively. The values of $T(t)$ were obtained by applying Equation S1 to the time-dependent upconverting emission spectra of the samples. Once the temperature profiles were recorded at distinct T_i, this procedure was performed to obtain thermal transients with the same range of temperature for comparison purposes.

A nonlinear noise reduction method based on the discrete wavelet transform (DWT) was used to reduce the noise arising from the θ(t) curves.[17] The denoising procedure was implemented through a custom MATLAB R2022a script in five steps, following a previously reported procedure.[15] The used script i) imports the thermometric parameter $\Delta = I_H/I_S$ from the as-measured temperature-dependent emission spectra; ii) computes the temperature by applying Equation S1 to the resulting time-dependent Δ; iii) converts the temperature into reduced temperature through Equation S4; iv) applies the DWT denoising method to obtain a denoised reduced temperature (threshold parameter: 15, 5 stages); and v) calculate the noise from the difference between the denoised and measured reduced temperature. The histograms of the noise obtained for all the denoised θ(t) curves result in a Gaussian distribution ($r^2 > 0.98$) centered at zero, following an additive white Gaussian noise that validates the denoising method herein applied.[18-19]

The denoised θ(t) curves were used to compute the critical onset time (t_i), which corresponds to the instant time at which the initial temperature starts to increase. After analyzing the data, the script marks t_i as the instant time at which the change in the denoised signal is higher than the standard deviation of the noise (extracted from the corresponding histogram). For the same initial temperature, the θ(t) curves were registered by irradiating the laser at distinct positions along the xx-direction (x_i = 0.0–6.0 mm, i = 1–4). The instantaneous ballistic Brownian velocity was then estimated as the slope of the x_i *versus* t_i plot, as demonstrated in **Figure S9**.

6. Calculation of crossover temperature

The value of the crossover temperature (T_c) was calculated from the intersection between the two straight lines adjusted to the bilinear trend of the temperature-dependent Brownian velocity of the UCNPs in the aqueous nanofluids. All possible combinations of two straight lines giving the best fit to the bilinear trend observed in the 300–350 K temperature range were computed by using a custom script written in MATLAB R2022a. The T_c values were then determined from the intersection between the two linear fits that give the maximum product of the coefficient of the determination (r^2) from each line. The uncertainty in T_c (δT_c) was estimated as:

$$\delta T_c = \frac{T_{cmax} - T_{cmin}}{2} = -\frac{(\sigma_{E2} + \sigma_{E1})}{m_1 - m_2} \quad \text{(S5)}$$

where T_{cmax} and T_{cmin} are the maximum and minimum predicted values of T_c, respectively, σ_{E1} and $\sigma_{E2,}$ and m_1 and m_2 are the standard error of the estimate (σ_E) and the slope (m) of the linear dependency below and above T_c, respectively. σ_E is defined as:

$$\sigma_E = \sqrt{\frac{\Sigma(\nu - \nu')^2}{n-2}} \quad \text{(S6)}$$

with ν and ν' corresponding to the measured and fitted values of the Brownian velocity, respectively, and n is the number of data points from each fitted line. The estimation of δT_c is illustrated in the schematic representation in **Figure S10**. The same procedure was employed regardless of the UCNPs' size or pH of the aqueous media.

7. Equipartition theorem

Equipartition theorem. The equipartition theorem describes the Brownian velocity as $\nu = \sqrt{k_B T/m^*}$, where k_B is the Boltzmann constant, T is the temperature, and m^* is the effective mass of the nanoparticles, representing the combined mass of UCNPs and half of the liquid mass moving cooperatively with them.[20] In this sense, dispersing UCNPs within denser solvents results in a lower ν, as shown in Figure 2a of the main text.

8. Brownian velocity and number of UCNPs per mL in the nanofluids

The number of UCNPs per unit of volume (V) was calculated considering the mass of the UCNPs in suspension

$$\frac{m_c}{V} = \rho_c \tag{S7}$$

where ρ_c is the mass concentration. The mass of each particle (assumed as a sphere with diameter d, TEM diameter, **Table S2**) is:

$$m_p = \rho_n \frac{\pi}{6} d^3 \tag{S8}$$

where ρ_n is the density of the UCNPs. The values of ρ_c, ρ_n, and d are presented in Table S1. The resulting number of UCNPs per unit of volume was estimated by:

$$\frac{N}{V} = \frac{m_c/V}{m_p} = \frac{6\rho_c}{\rho_n \pi d^3} \tag{S9}$$

and is presented in Table S4. Plotting the Brownian velocity at 300 K as a function of the diameter and number of UCNPs per mL, results in the plots presented in **Figure S10**.

We notice the increase in the Brownian velocity with the increase of the number of UCNPs, in line with what was reported by us before, stressing the pivotal role of particle-particle interactions in the measured Brownian velocity.[14]

9. Supplementary Figures

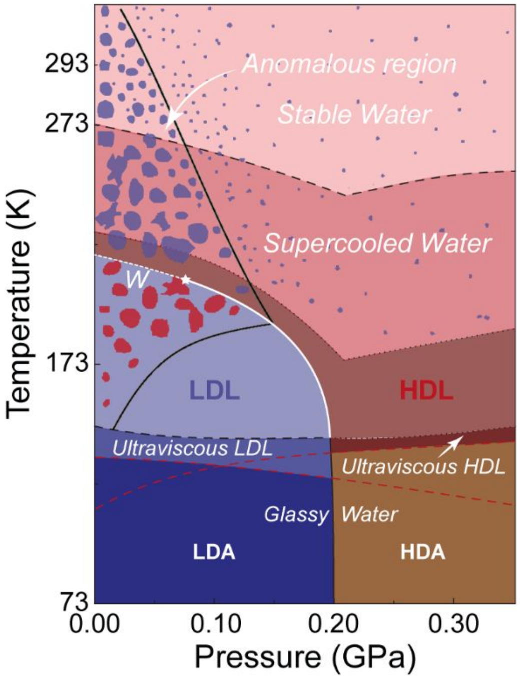

Figure S1. Hypothetical phase diagram of liquid water showing the coexistence of LDL (blue) and HDL (red) domains near the W line. Below W, LDL dominates with fluctuations in HDL domains, whereas above W HDL dominates with LDL fluctuations. The white star represents the liquid-liquid critical point. As one moves away from the critical point, the fluctuations decrease in size, as evidenced by the diminishing size of the blobs. The black line delimits the so-called "funnel of life" at which water exhibits its unusual properties that are crucial for sustaining life. Reproduced from Pettersson, L. G. M. In A two-state picture of water and the funnel of life, Modern Problems of the Physics of Liquid Systems, L.A., B.; L., X., Eds. Springer Proceedings in Physics: 2019; pp 3-39. Copyright 2019 Springer Nature.

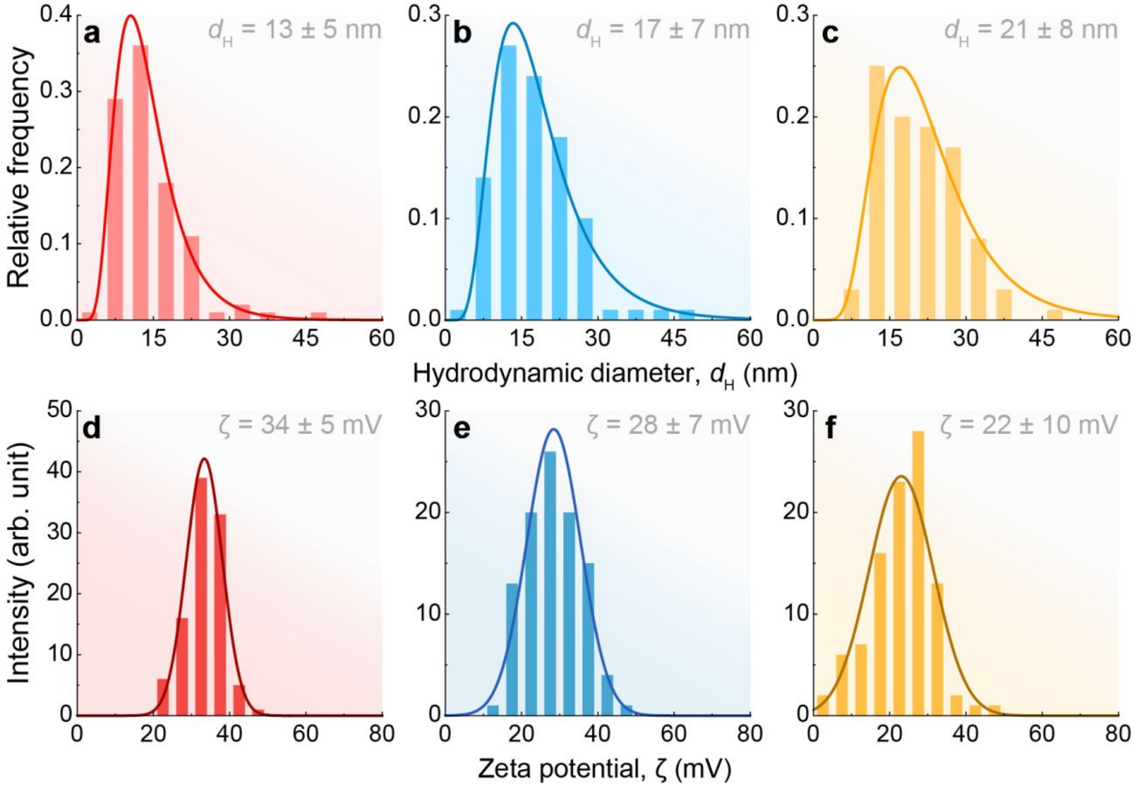

Figure S2. Hydrodynamic diameter (d_H, top) and ζ (bottom) of the 15 nm nanofluids prepared in **(a, d)** water (pH = 5.10), **(b, e)** heavy water, and **(c, f)** ethanol. The lines are the best fits to the data using lognormal (d_H) and Gaussian (ζ) functions ($r^2 > 0.97$). The values of d_H obtained for the other nanofluids prepared in water are summarized in Table S2.

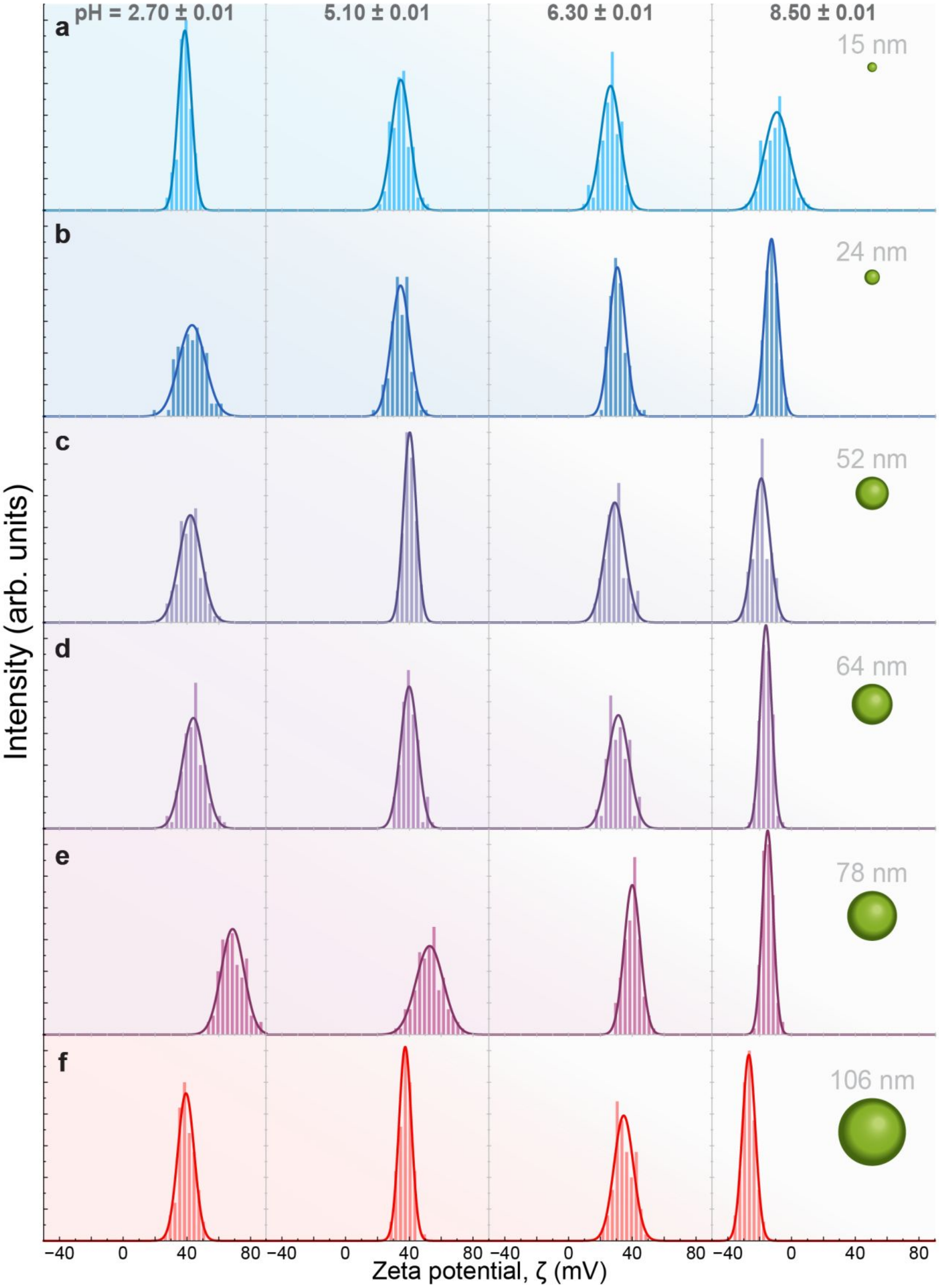

Figure S3. Zeta potentials of the aqueous nanofluids containing UCNPs with **(a)** 15, **(b)** 24, **(c)** 52, **(d)** 64, **(e)** 78, and **(f)** 106 nm in diameter measured at four distinct pH values. The lines are the best fits for ζ (Gaussian distribution, $r^2 > 0.98$). The fitting results (ζ values and corresponding uncertainties) are shown in Figure S4. For the studied volume fraction, the 100 nm UCNP with a silica shell is only stable at pH = 5.10, at which $\zeta = -33 \pm 11$ mV.

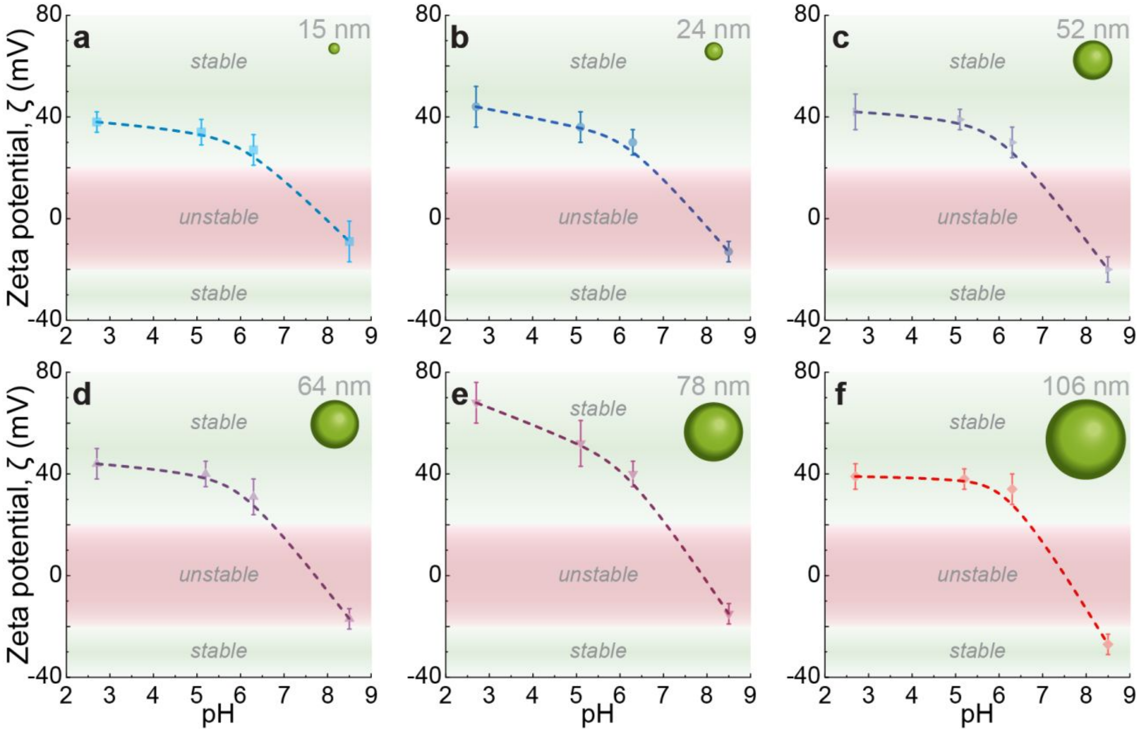

Figure S4. pH dependence of ζ for the **(a)** 15 nm, **(b)** 24 nm, **(c)** 52 nm, **(d)** 64 nm, **(e)** 78 nm, and **(f)** 106 nm UCNPs dispersed in water, obtained from the data in Figure S3. The lines are guides for the eyes. The decrease in ζ with the increase in pH is in good accordance with previous reports,[21] highlighting how the presence of ions in the medium can affect the surface charge of UCNPs. The stability of a colloid is the result of van der Waals attraction and repulsion (steric and electrostatic). The zeta potential provides information about the repulsive forces due to the electric double layer and, thus, the absolute values, |ζ|, measure the magnitude of the electrostatic repulsion. The nanofluids are considered stable if $|\zeta| \geq 20$ mV (green-shaded regions).[22]

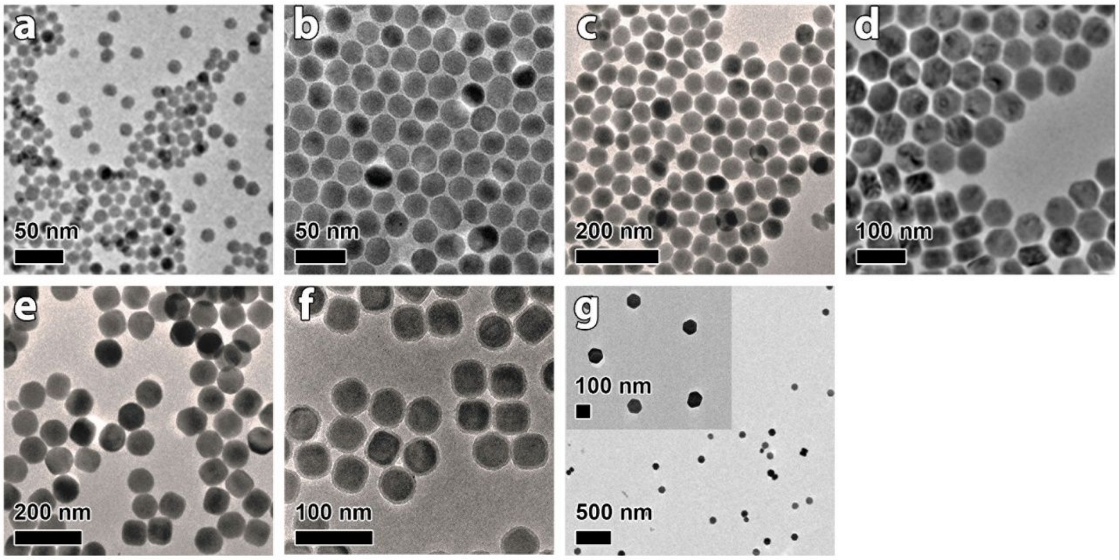

Figure S5. TEM micrographs of **(a)** 15 nm, **(b)** 24 nm, **(c)** 52 nm, **(d)** 64 nm, **(e)** 78 nm, **(f)** 100 nm (core = 78 nm and a SiO_2 shell of 11 nm thick), and **(g)** 106 nm ligand-free UCNPs.

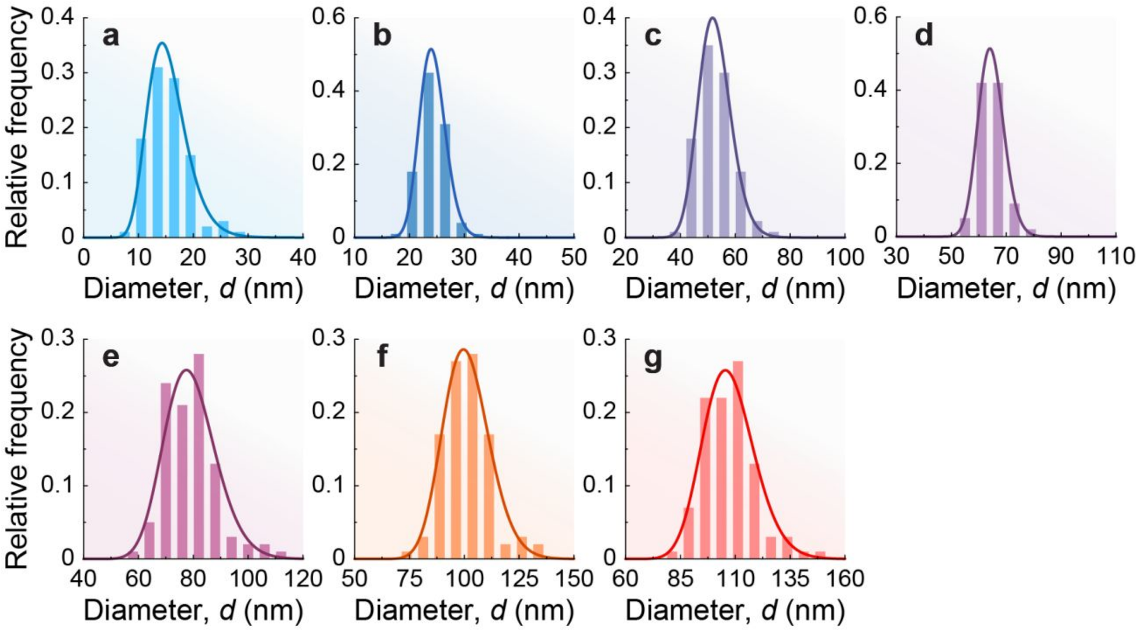

Figure S6. TEM size distribution of the **(a)** 15, **(b)** 24, **(c)** 52, **(d)** 64, **(e)** 78, **(f)** 100 nm (SiO_2 shell), and **(g)** 106 nm ligand-free UCNPs. The lines are the best fits for TEM size data (Figure S5) using a lognormal distribution (r^2 > 0.97). Fitting results (diameter *d* and corresponding uncertainty) are presented in Table S2.

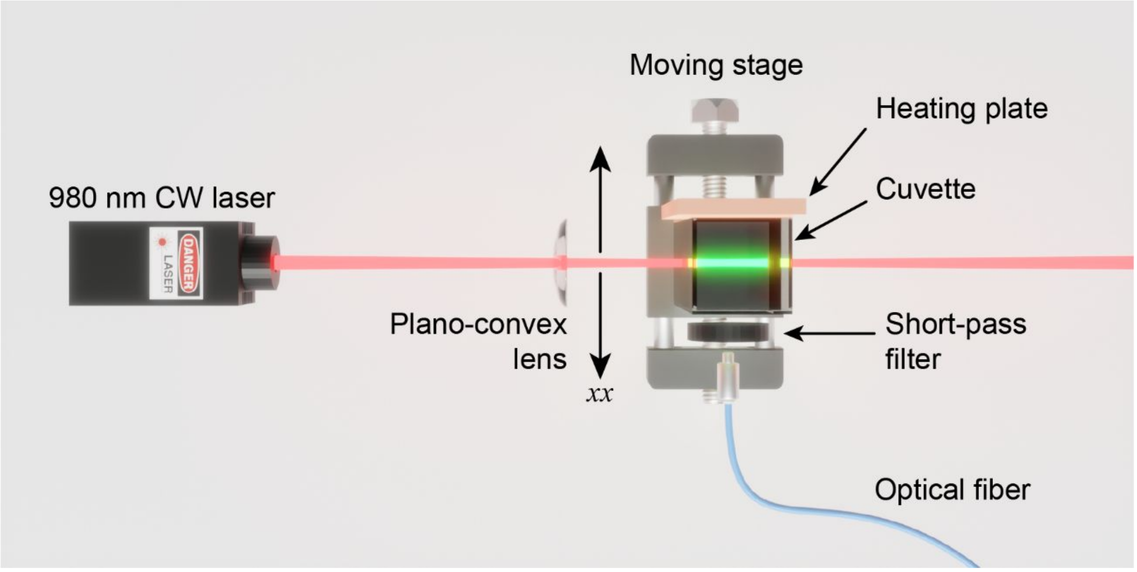

Figure S7. Schematic of the experimental setup used to record the thermal transients of the nanofluids. The cuvette is placed on a controlled moving stage that allows the nanofluid to be irradiated at different positions along the *xx* direction. The 980 nm laser beam is collimated by a plano-convex lens and the light emission from the nanofluid is collected by an optical fiber coupled to a portable spectrometer.

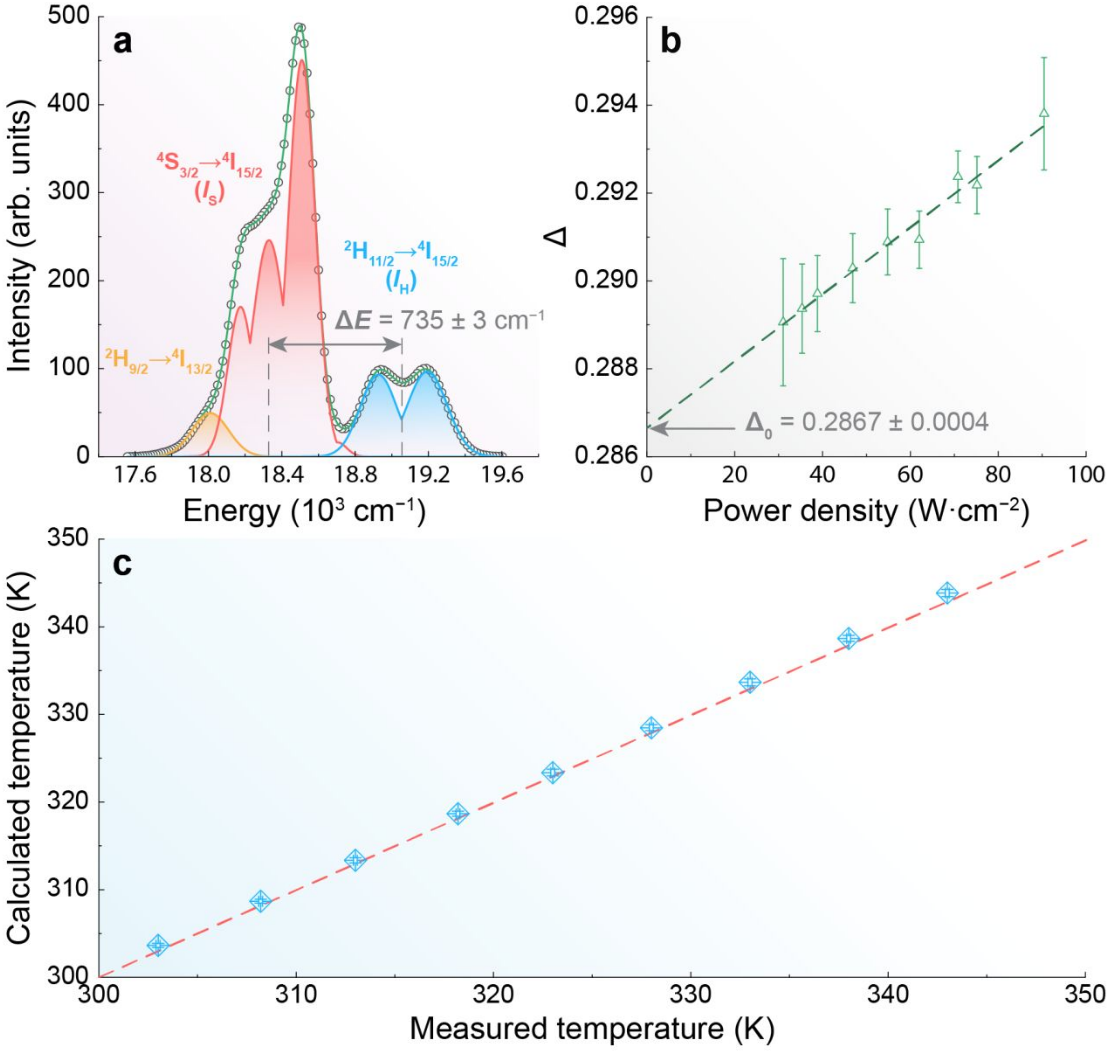

Figure S8. An illustrative example of thermal calibration from the 15 nm aqueous nanofluid (pH = 5.10). **(a)** Determination of the energy separation ΔE at 303.0 K. The areas shaded in yellow, red, and blue are the integrated intensities of the $^2H_{9/2} \rightarrow {}^4I_{13/2}$, $^4S_{3/2} \rightarrow {}^4I_{15/2}$ (I_S), and $^2H_{11/2} \rightarrow {}^4I_{15/2}$ (I_H) transitions of Er^{3+}. The green solid line is the fit envelope while the grey circles are the data from the emission spectrum measured at 303 K under excitation at 980 nm (62·W cm^{-2}). **(b)** Power dependence on the thermometric parameter Δ. The dashed green line is the best fit of the experimental data to a straight line ($r^2 > 0.99$), allowing to determine the value of Δ_0 at T_0 = 300.0 ± 0.1 K. **(c)** Temperature calculated from Equation S1 (x-axis) *versus* the temperature measured with a K-type thermocouple (y-axis). The dashed red line is a guide for the eyes corresponding to $x = y$.

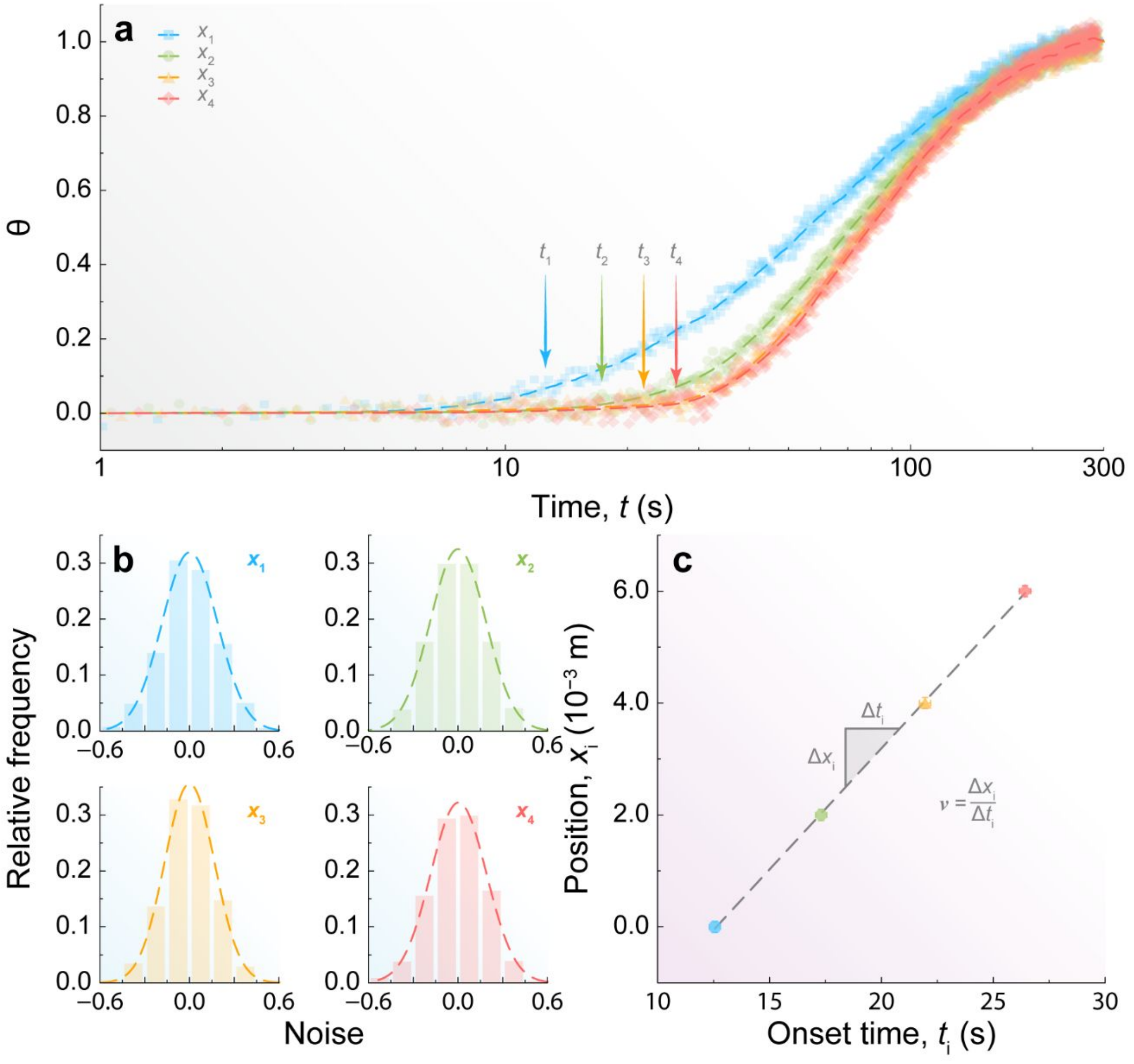

Figure S9. Determination of the instantaneous Brownian velocity of 15 nm UCNPs dispersed in water (pH = 5.10 ± 0.01). **(a)** Illustrative denoising procedure applied to four reduced temperatures (symbols) recorded at positions x_1 = 0.0 mm, x_2 = 2.0 mm, x_3 = 4.0 mm, and x_4 = 6.0 mm. The dashed lines are the denoised signal obtained through the DWT procedure, where the onset t_i values are indicated. **(b)** Histograms of the noise curves in panel **(a)**. The dashed lines are the best fits to experimental data using Gaussian functions. It is possible to observe that the noise is nearly centered at zero, presenting a high coefficient of determination (r^2 > 0.98) for all samples, indicating that it corresponds to an additive Gaussian noise. **(c)** Determination of the instantaneous Brownian velocity v, corresponding to the slope ($\Delta x_i/\Delta t_i$) of the straight line adjusted to the experimental data from panel **(a)**. The error bars in x_i and t_i are the uncertainty in the position of the moving stage from Figure S7 (0.1 mm) and the integration time used for spectral acquisition (250 ms), respectively.

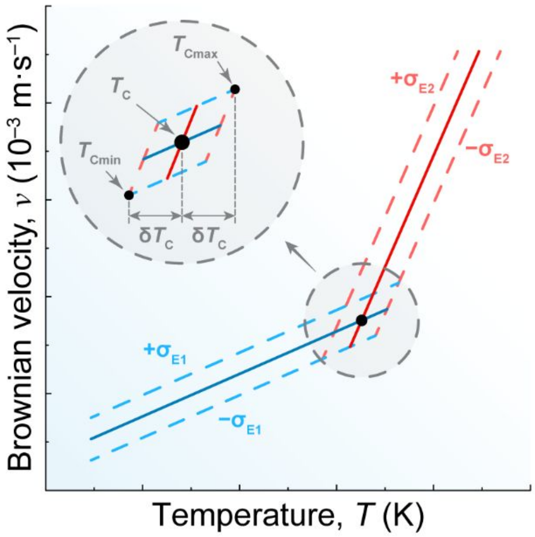

Figure S10. Schematic representation of the calculation of the crossover temperature and its uncertainty ($T_c \pm \delta T_c$). The solid blue and red lines represent linear fits to the experimental data before and after T_c, respectively. The corresponding dashed lines depict the average deviation in the prediction of the Brownian velocity.

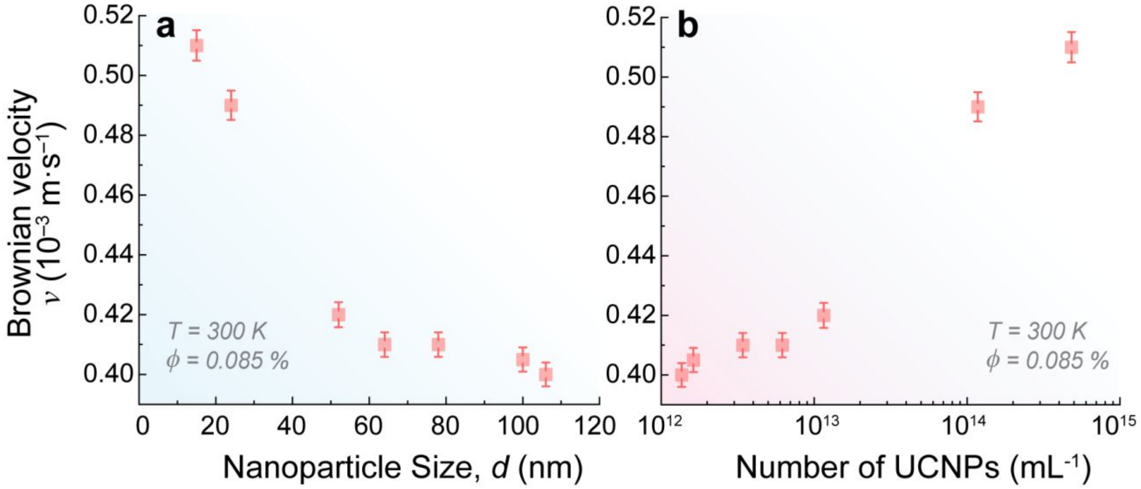

Figure S11. Brownian velocity of the UCNPs in the aqueous nanofluids. The Brownian velocity is represented as a function of **(a)** the particle size and **(b)** the number of UCNPs per mL at 300 K and pH=5.10 (illustrative temperature and pH values).

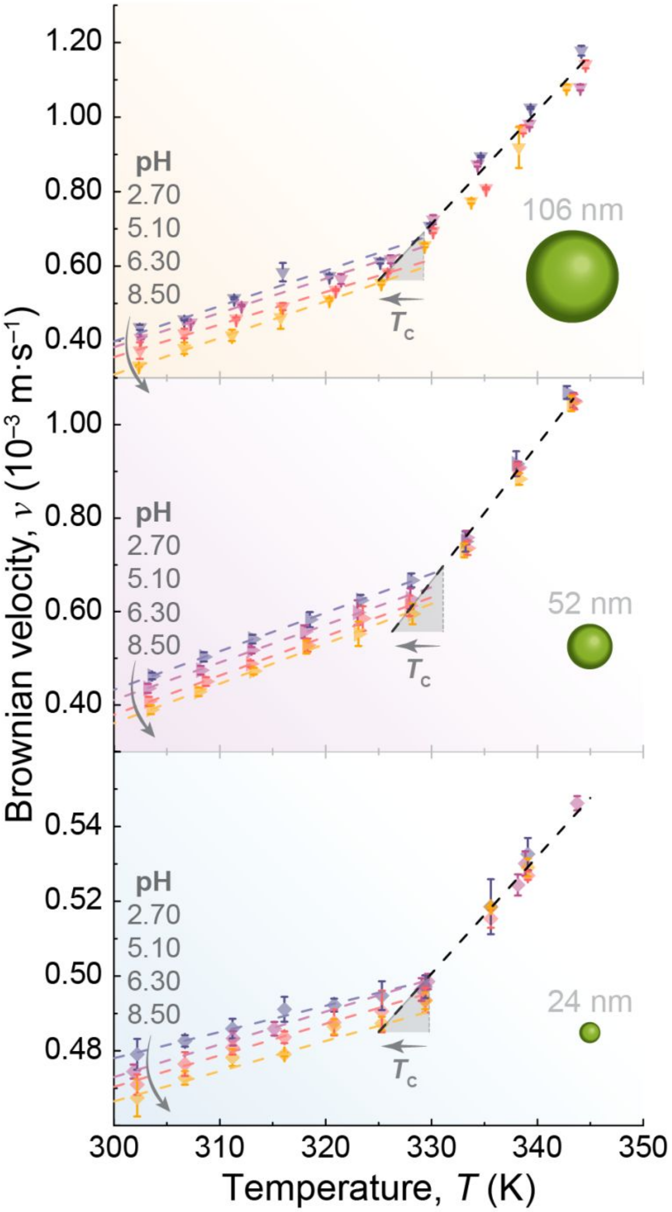

Figure S12. Effect of pH on Brownian velocity of 24 nm, 52 nm, and 106 nm UCNPs. The lines are the best linear fits at each pH for $T<T_c$ and the same linear fit for all the pH values for $T>T_c$ ($r^2>0.98$ for all samples). The pH-dependent variation in the Brownian velocity of the 100 nm UCNPs with a silica shell was not assessed due to their lower colloidal stability for pH values different than 5.10.

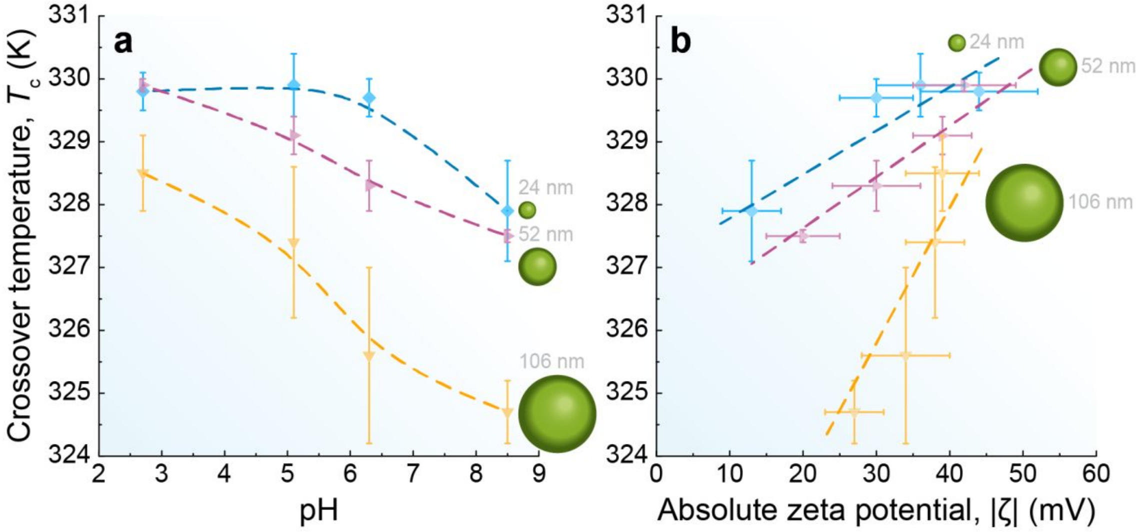

Figure S13. Crossover temperature as a function of the **(a)** pH and **(b)** |ζ| for the 24, 52, and 106 nm diameter UCNPs. The lines are guides for the eyes.

10. Supplementary Tables

Table S1. Size, chemical compositions of the core and shell, density (ρ_n), mass concentration (ρ_c), and volume fraction ($\phi = \rho_c/\rho_n$) of UCNPs dispersed in EtOH, D_2O, and H_2O.

Solvent	Size (nm)	Core	Shell	ρ_n ($kg\cdot m^{-3}$)	ρ_c ($kg\cdot m^{-3}$)	ϕ (%)
EtOH<br>D_2O<br>H_2O	15	$NaGdF_4$:Yb/Er(18/2%)	$NaGdF_4$	5,653	4.81	
H_2O	24	$NaYF_4$:Yb/Er(18/2%)	$NaYF_4$	4,384	3.73	
	52	$NaYF_4$:Lu/Yb/Er(40/18/2%)	-	5,456	4.64	0.085
	64	$NaYF_4$:Lu/Yb/Er(47/18/2%)	-	5,616	4.77	
	78	$NaYF_4$:Lu/Yb/Er(47/18/2%)	-	5,616	4.77	
	100	$NaYF_4$:Lu/Yb/Er(47/18/2%)	SiO_2	3,821	3.25	
	106	$NaYF_4$:Lu/Yb/Er(50/18/2%)	-	5,685	4.83	

Table S2. TEM (d) and hydrodynamic (d_H) diameters of the obtained UCNPs. The values of hydrodynamic diameters from DLS measurements correspond to the water-based nanofluids at pH = 5.10 ± 0.01. An excellent agreement was observed for the mean diameters reported by TEM and DLS.

UCNP	TEM diameter, d (nm)	Hydrodynamic diameter, d_H (nm)
$NaGdF_4$:Yb/Er(18/2%)@$NaGdF_4$	15 ± 3	13 ± 5
$NaYF_4$:Yb/Er(18/2%)@$NaYF_4$	24 ± 2	36 ± 8
$NaYF_4$:Lu/Yb/Er(40/18/2%)	52 ± 5	50 ± 7
$NaYF_4$:Lu/Yb/Er(47/18/2%)	64 ± 4	60 ± 8
$NaYF_4$:Lu/Yb/Er(47/18/2%)	78 ± 8	80 ± 12
$NaYF_4$:Lu/Yb/Er(47/18/2%)@SiO_2	100 ± 9	90 ± 22
$NaYF_4$:Lu/Yb/Er(50/18/2%)	106 ± 10	93 ± 26

Table S3. Energy separation (ΔE), maximum S_r (S_m), minimum temperature uncertainty (δT), and corresponding temperature at which they occur for each nanofluid.

Solvent	Size (nm)	ΔE (cm^{-1})	S_m ($\%\cdot K^{-1}$)	δT (K)	T (K)
EtOH	15	735 ± 3	1.15	0.6	303.0 ± 0.1
D_2O				0.3	
H_2O				0.4	
H_2O	24	680 ± 11	1.07	0.5	
	52	722 ± 9	1.13	0.4	
	64	740 ± 12	1.16	0.4	
	78	688 ± 8	1.08	0.5	
	100	688 ± 8	1.08	0.5	
	106	699 ± 10	1.10	0.5	

Table S4. Number of UCNPs dispersed in 1 mL of the investigated aqueous nanofluids (N/V) for a volume fraction of 0.085%.

UCNP	TEM diameter, d (nm)	N/V (mL^{-1})
$NaGdF_4$:Yb/Er(18/2%)@$NaGdF_4$	15	4.8×10^{14}
$NaYF_4$:Yb/Er(18/2%)@$NaYF_4$	24	1.2×10^{14}
$NaYF_4$:Lu/Yb/Er(40/18/2%)	52	1.2×10^{13}
$NaYF_4$:Lu/Yb/Er(47/18/2%)	64	6.2×10^{12}
$NaYF_4$:Lu/Yb/Er(47/18/2%)	78	3.4×10^{12}
$NaYF_4$:Lu/Yb/Er(47/18/2%)@SiO_2	100	1.6×10^{12}
$NaYF_4$:Lu/Yb/Er(50/18/2%)	106	1.4×10^{12}

11. References

1. Poole, P. H.; Sciortino, F.; Essmann, U.; Stanley, H. E. Phase-behavior of metastable water. *Nature* **1992,** *360* (6402), 324-328, DOI: 10.1038/360324a0.
2. Neophytou, A.; Chakrabarti, D.; Sciortino, F. Topological nature of the liquid–liquid phase transition in tetrahedral liquids. *Nat. Phys.* **2022,** *18* (10), 1248-1253, DOI: 10.1038/s41567-022-01698-6.
3. Debenedetti, P. G.; Sciortino, F.; Zerze, G. H. Second critical point in two realistic models of water. *Science* **2020,** *369* (6501), 289-292, DOI: 10.1126/science.abb9796.
4. Fanetti, S.; Lapini, A.; Pagliai, M.; Citroni, M.; Di Donato, M.; Scandolo, S.; Righini, R.; Bini, R. Structure and dynamics of low-density and high-density liquid water at high pressure. *J. Phys. Chem. Lett.* **2014,** *5* (1), 235-240, DOI: 10.1021/jz402302z.
5. Oka, K.; Shibue, T.; Sugimura, N.; Watabe, Y.; Tanaka, M.; Winther-Jensen, B.; Nishide, H. Two states of water converge to one state below 215 K. *J. Phys. Chem. Lett.* **2021,** *12* (24), 5802-5806, DOI: 10.1021/acs.jpclett.1c01132.
6. Kringle, L.; Thornley, W. A.; Kay, B. D.; Kimmel, G. A. Reversible structural transformations in supercooled liquid water from 135 to 245 K. *Science* **2020,** *369* (6510), 1490-1492, DOI: 10.1126/science.abb7542.
7. Suzuki, Y. Experimental estimation of the location of liquid-liquid critical point for polyol aqueous solutions. *J Chem Phys* **2018,** *149* (20), 204501, DOI: 10.1063/1.5050832.
8. Suzuki, Y. Direct observation of reversible liquid-liquid transition in a trehalose aqueous solution. *Proc. Natl. Acad. Sci. USA* **2022,** *119* (5), e2113411119, DOI: 10.1073/pnas.2113411119.
9. Kim, K. H.; Amann-Winkel, K.; Giovambattista, N., et al. Experimental observation of the liquid-liquid transition in bulk supercooled water under pressure. *Science* **2020,** *370* (6519), 978-982, DOI: 10.1126/science.abb9385.
10. Pettersson, L. G. M. In *A two-state picture of water and the funnel of life*, Modern Problems of the Physics of Liquid Systems, L.A., B.; L., X., Eds. Springer Proceedings in Physics: 2019; pp 3-39.
11. Rosu-Finsen, A.; Davies, M. B.; Amon, A.; Wu, H.; Sella, A.; Michaelides, A.; Salzmann, C. G. Medium-density amorphous ice. *Science* **2023,** *379* (6631), 474-478, DOI: 10.1126/science.abq2105.
12. Wang, F.; Han, Y.; Lim, C. S., et al. Simultaneous phase and size control of upconversion nanocrystals through lanthanide doping. *Nature* **2010,** *463* (7284), 1061-1065, DOI: 10.1038/Nature08777.
13. Wu, Y.; Xu, J.; Qin, X.; Xu, J.; Liu, X. Dynamic upconversion multicolour editing enabled by molecule-assisted opto-electrochemical modulation. *Nat. Commun.* **2021,** *12* (1), 2022, DOI: 10.1038/s41467-021-22387-7.
14. Brites, C. D.; Xie, X.; Debasu, M. L., et al. Instantaneous ballistic velocity of suspended Brownian nanocrystals measured by upconversion nanothermometry. *Nat. Nanotechnol.* **2016,** *11* (10), 851-856, DOI: 10.1038/nnano.2016.111.
15. Brites, C. D. S.; Zhuang, B. L.; Debasu, M. L., et al. Decoding a percolation phase transition of water at ~330 k with a nanoparticle ruler. *J. Phys. Chem. Lett.* **2020,** *11* (16), 6704-6711, DOI: 10.1021/acs.jpclett.0c02147.
16. Balabhadra, S.; Debasu, M. L.; Brites, C. D. S.; Ferreira, R. A. S.; Carlos, L. D. Upconverting nanoparticles working as primary thermometers in different media. *J. Phys. Chem. C* **2017,** *121* (25), 13962-13968, DOI: 10.1021/acs.jpcc.7b04827.

17. Lang, M.; Guo, H.; Odegard, J. E.; Burrus, C. S.; Wells, R. O. Nonlinear processing of a shift-invariant discrete wavelet transform (DWT) for noise reduction. In *Wavelet applications ii,* , Szu, H. H., Ed. Proceedings SPIE: 1995; Vol. 2491, pp 640-651.
18. Donoho, D. L.; Johnstone, I. M. Ideal spatial adaptation by wavelet shrinkage. *Biometrika* **1994,** *81* (3), 425-455, DOI: 10.1093/biomet/81.3.425.
19. Donoho, D. L. De-noising by soft-thresholding. *IEEE T. Inform. Theory* **1995,** *41* (3), 613-627, DOI: 10.1109/18.382009.
20. Huang, R. X.; Chavez, I.; Taute, K. M.; Lukic, B.; Jeney, S.; Raizen, M. G.; Florin, E. L. Direct observation of the full transition from ballistic to diffusive brownian motion in a liquid. *Nat. Phys.* **2011,** *7* (7), 576-580, DOI: 10.1038/Nphys1953.
21. Bogdan, N.; Vetrone, F.; Ozin, G. A.; Capobianco, J. A. Synthesis of ligand-free colloidally stable water dispersible brightly luminescent lanthanide-doped upconverting nanoparticles. *Nano Lett.* **2011,** *11* (2), 835-840, DOI: 10.1021/nl1041929.
22. Bhattacharjee, S. DLS and zeta potential - What they are and what they are not? *J. Control Release* **2016,** *235*, 337-351, DOI: 10.1016/j.jconrel.2016.06.017.
