# Supplementary material for: Deciphering Density Fluctuations in the Hydration Water of Brownian Nanoparticles via Upconversion Thermometry
Source: J Phys Chem Lett. 2024 Feb 29;15(9):2606–15. doi: 10.1021/acs.jpclett.4c00044 (PMC10926164; doi:10.1021/acs.jpclett.4c00044)
Supplement: Supplementary file 2 — jz4c00044_si_002.pdf [file jz4c00044_si_002.pdf]

jz-2024-000448.R1

Name: Peer Review Information for "Deciphering Density Fluctuations in the Hydration Water of Brownian Nanoparticles via Upconversion Thermometry"

First Round of Reviewer Comments

Reviewer: 1

Comments to the Author

Report on manuscript jz-2024-000448

The paper presents interesting experimental results on the Brownian motion of nanoparticles of various sizes in three different liquids (H_2O, D_2O and ethanol) followed using upconversion thermometry. The data are intriguing and should definitely be published. I find the measured difference between H_2O and D_2O quite unexpected and thus of particular interest. However, there are some more speculative interpretations that I would recommend the authors to reconsider. I recommend publication after the authors have considered the specific comments below:

1) Page 5, line 10. The distinguishing feature between continuum models of liquid water and the two-state model is not a unimodal density. In the two-state, liquidliquid critical point picture ambient liquid water is a supercritical phase as it exists in the one-phase region beyond a critical point. It is still a single phase, but with fluctuations on some time- and length-scale that on average give a unimodal density distribution. I would rather emphasize that in the continuum models fluctuations are assumed to be thermal in character, while in the two-state model(s) structural fluctuations begin to contribute around the compressibility minimum.

2) Page 8, Figure caption 1, line 17. The second “while” is superfluous.

3) Page 11, Figure 2a. The large difference in behavior between H_2O and D_2O needs a deeper analysis and discussion. Typically, the physical and thermodynamical properties are the same for the two, albeit with a temperature offset of ~5 K. E.g., the viscosity which could be of importance for the Brownian motion, has a scaling by 1.0544 and an offset of -6.498 K compared to H_2O. Nuclear quantum effects have been found to be independent of temperature (Kim et al., PRL **119**, 075502 (2017)). The present reviewer thus finds the observed difference in behavior of D_2O (strictly linear) and H_2O (bilinear) very surprising! The authors refer to the work of Roke and

coworkers (ref. 76), but this study was on the effects of 3D confinement. This should be made clear and discussed in the discussion on page 14. Are the effects of confinement inside liposomes transferrable to the outside of the nanoparticles?

4) Page 13, line 44. Note that the Widom line is shifted by a mere 4 K in ref. 32. The displacement of the density maximum and the compressibility minimum are also small. The authors do need to reconsider the statement that such effects would shift D_2O beyond the liquid phase!

5) Page 17, line 27. The comparison of S/V ratios is misleading. The volume goes as the third power and the surface as the second, so the relationship will be linear. Obviously, S/V will increase by a greater amount when comparing spheres that differ more in size. There is nothing special about these values.

6) Page 20, lines 20-26. The statement that "Increasing pH triggers the fragmentation of LDL domains into smaller ones" needs a reference or something to substantiate the claim.

7) Page 20, line 34. "As pressure increases…there is a fragmentation of LDL domains" needs a reference or data to support this statement. Do they fragment or become less favorable?

8) Page 21, lines 44-54. The reason why more thermal energy required for HDL to LDL fluctuations should result in more LDL domains needs to be given. As stated, it would seem that the result should be less LDL, not more.

9) Page 23, lines 13-17. "Additionally, we have shown that increasing pH disrupts the tetrahedral organization". This has not been shown in the present work. It is a speculation.

10) Page 23, lines 44-47. "Invaluable for experimentally quantifying HDL/LDL proportions in the hydration water". The authors have shown interesting differences that they associate (as a hypothesis) with conversion between HDL and LDL, but there has not been any attempt to *quantify* the fractions. The statement is thus poorly supported by the data that is presented.

11) SI, Point 6. The conversion between HDL and LDL has been experimentally observed in ref 37 (main paper) which could be stronger evidence than Poole et al.

12) Figure S9b. Noise histograms (8 classes)? I only see 4 histograms.

Reviewer: 2

Comments to the Author

This is a very interesting work in which authors investigate the variation of the cross over temperature of water as a function of the particel size used for sensing and as a function of the pH. Autthors use upconverting nanoparticles for determining the brownian velocity and how this brownian velocity changes with temperature. The crossover temperature (that determines the temperature at which equlibrium between high density and low density domians is broken) is determined by analyzing the two linear regimes that are obtained in the plot of brownian velocity vs temperature. The main conclusion, if I understood well the paper, is that the as the particle size is reduced the presence of high density domains is reduced and the critical temperature increases as the particel size decreases. I think this conclusion is valuable and reasonable. The question here is that authors did not abserve any net change of the brownian velocity with particle size. For sizes between 50 and 100 nm the brownian velocity seems to be independent on size. This sounds strange for me. Is there any easy explanation for this?

Authors also studied the change of the crossover temperature with the pH. Again I think that the reasoning given by authors is good but then I have some difficulties to understand why the results here obtined do not agree with previous papers also dealing with the variation of the crossover temperature with the pH. By the use of optical twezzers or by using the luminescence of certain complexes it has been found that crossover temperature increases with the pH. But data in Figure 4 seems to indicate that in these experiments the tendency is opposite. I think that this point should be discussed in detail in a revised version.

In summary, this is a hot topic, authors are a reference in the field and the paper is well written and explained. But I think there are two critical points (described above) that need to be considered before the paper can be accepted.

Author's Response to Peer Review Comments:

Prof. Juan Bisquert
Senior Editor
The Journal of Physical Chemistry Letters

Prof. Dr. Luís António Dias Carlos
UNIVERSIDADE DE AVEIRO
DEPARTAMENTO DE FÍSICA
3810-193 AVEIRO, PORTUGAL
Phone +351 234370946
lcarlos@ua.pt
http://hybrids.web.ua.pt/LDCarlos.html
http://hybrids.web.ua.pt/index.html

February 08, 2024

Dear Prof. Juan Bisquert,

I would like to bring to your consideration the revised version of the manuscript **jz-2024-000448n-2022-05625s** entitled ***"Deciphering density fluctuations in the hydration water of Brownian nanoparticles via upconversion thermometry"*** by F. E. Maturi, R. S. R. Filho, C. D. S. Brites, J. Fan, R. He, B. Zhuang, X. Liu, and L. D. Carlos, for publication in *The Journal of Physical Chemistry Letters.*

We appreciated reading the positive and thoughtful reviews of our manuscript. We have carefully considered the reviewers' concerns modifying the manuscript to address all of them. For you and the reviewers to see the changes more easily, we have included a revised version of both the manuscript and Supporting Information where the modified texts are highlighted in yellow.

The ***point-by-point response to reviewers*** is shown below where excerpts from the original reports of the reviewers are italicized and our replies are marked in blue. We believe the revisions, including the supplementary data and additional analyses, fully address the concerns raised.

Thank you again for your time and effort in considering our manuscript.

On behalf of all the authors, please accept my best regards.
We look forward to hearing from you soon.

Sincerely,

(Full Professor)

Point-by-Point Reply to Reviewers

Reviewer #1

The paper presents interesting experimental results on the Brownian motion of nanoparticles of various sizes in three different liquids (H_2O, D_2O and ethanol) followed using upconversion thermometry. The data are intriguing and should definitely be published. I find the measured difference between H_2O and D_2O quite unexpected and thus of particular interest. However, there are some more speculative interpretations that I would recommend the authors to reconsider. I recommend publication after the authors have considered the specific comments below:

We appreciate the valuable insights and constructive comments provided by the reviewer, which have been crucial in elevating the scholarly merit of our work. After recognizing the presence of a few speculative interpretations in the original manuscript, we have thoroughly worked to eliminate ambiguity to enhance clarity in the revised version. Every suggestion has been considered, leading to a series of revisions aiming to address the reviewer's comments and suggestions, as observed below.

1) Page 5, line 10. The distinguishing feature between continuum models of liquid water and the two-state model is not a unimodal density. In the two-state, liquid-liquid critical point picture ambient liquid water is a supercritical phase as it exists in the one-phase region beyond a critical point. It is still a single phase, but with fluctuations on some time- and length-scale that on average give a unimodal density distribution. I would rather emphasize that in the continuum models fluctuations are assumed to be thermal in character, while in the two-state model(s) structural fluctuations begin to contribute around the compressibility minimum.

Reply

We acknowledge the reviewer's observation. The sentence was improved and modified in the revised version.

2) Page 8, Figure caption 1, line 17. The second "while" is superfluous.

Reply

This suggestion was implemented in the revised version of the manuscript.

3) Page 11, Figure 2a. The large difference in behavior between H_2O and D_2O needs a deeper analysis and discussion. Typically, the physical and thermodynamical properties are the same for the two, albeit with a temperature offset of ~5 K. E.g., the viscosity which could be of importance for the Brownian motion, has a scaling by 1.0544 and an offset of -6.498 K compared to H_2O. Nuclear quantum effects have been found to be independent of temperature (Kim et al., PRL 119, 075502 (2017)). The present reviewer thus finds the observed difference in behavior of D_2O (strictly linear) and H_2O (bilinear) very surprising! The authors refer to the work of Roke and coworkers (ref. 76), but this study was on the effects of 3D confinement. This should be made clear and discussed in the discussion on page 14. Are the effects of confinement inside liposomes transferrable to the outside of the nanoparticles?

Reply

As emphasized by the reviewer, the thermodynamical properties of H_2O and D_2O typically exhibit a temperature offset of ~+5 K, as observed in the melting point (+4 K), maximum density (+7 K), isothermal compressibility (+5 K), nuclear quantum effects (+5 K, *Phys. Rev. Lett.* 119, 075502, **2017**), viscosity (–6.5 K, *J. Chem. Eng. Data* 49.4, 1064-1069, **2004**), and the Widom line at ~0 bar (+4 K, *Science* 358, 1589-1593, **2017**).

Accordingly, we expected to observe a bilinear trend with a similar shift in the temperature-dependent Brownian velocity of the UCNPs when suspended in D_2O. However, the D_2O data revealed a strict linearity, which deviated from our expectations (Figure 2a of the submitted manuscript) and was equally surprising to us.

We believe that the work of Roke et al. (Ref. 76 in the revised manuscript) is relevant to discuss this discrepancy between H_2O and D_2O results and we thank the reviewer for bringing up this discussion.
In that work, the authors demonstrated that, under 3D confinement, H_2O exhibits a longer-ranged ordered structure while D_2O does not, revealing that H_2O molecules influence each other to a much larger spatial extent than D_2O. Consequently, the resulting H-bond networks in H_2O and D_2O differ from intermolecular length scales up to ~100 nm length scales. The pertinence of whether these findings are related to our results or not is an insightful question.
For that, we would like to stress two key points:

- Experimental (*Science* 360, 1339, **2018**) and simulation (*J. Chem. Phys.* 111, 1223, **1999**; *J. Phys. Chem. B* 105, 5106, **2001**) results have already shown that the dielectric constant of confined water is much lower than bulk water. Similarly, the dielectric constant of interfacial water in the electric double layer is distorted and also much lower than that of bulk water (*Nat. Commun.* 13, 3742, **2022**). This distortion arises from a complex interplay between the NPs' surface with their nanoenvironment, as demonstrated by Kim et al. (*J. R. Soc. Interface* 11, 20130931, **2014**). Interestingly, these results demonstrate how the temperature can be used to change the dielectric constant at the hydration layer by mitigating the effect of surface charge at water-oil interfaces because, at a higher temperature, the confinement of interfacial water molecules by surface charge diminishes, leading to an increased permittivity (*Nat. Commun.* 13, 3742, **2022**).
- It is important to point out that the length scale over which the confinement effects are observed (> 100 nm corresponding to more than 2×10^7 H_2O molecules) by Roke et al. is considerably larger than the confinement length scales that have been previously observed, of approximately 2-20 nm (Ref. 76 in the revised manuscript).

A new sentence was added to the revised manuscript to include this discussion.

4) Page 13, line 44. Note that the Widom line is shifted by a mere 4 K in ref. 32. The displacement of the density maximum and the compressibility minimum are also small. The authors do need to reconsider the statement that such effects would shift D_2O beyond the liquid phase!

Reply

We agree with the reviewer. This sentence is speculative and was removed in the revised version of the manuscript. As discussed in the previous question, a temperature shift of a few degrees is typically observed in the thermodynamic properties of D_2O in comparison to those of H_2O. Thus, it is speculative to argue that T_c can potentially reside beyond the confines of the liquid phase due to that shift in the thermodynamic properties of D_2O. In the revised version, we also added a short sentence with the temperature offset values between the thermodynamical properties of H_2O and D_2O mentioned in the first part of the reply to the previous question.

5) Page 17, line 27. The comparison of S/V ratios is misleading. The volume goes as the third power and the surface as the second, so the relationship will be linear. Obviously, S/V will increase by a greater amount when comparing spheres that differ more in size. There is nothing special about these values.

Reply

The reviewer is indeed right because representing the crossover temperature (T_c) as a function of either the particle diameter (d) or surface-to-volume ratio (S/V) yields an identical analysis outcome: T_c increases while decreasing the particle size. Therefore, the inset in Figure 2c merely displays the data differently. Therefore, the discussion concerning the S/V values has been omitted in the revised manuscript to prevent any potential misinterpretation. Nevertheless, we kept the inset in Figure 2c because it is more visually appealing.

6) Page 20, lines 20-26. The statement that “Increasing pH triggers the fragmentation of LDL domains into smaller ones” needs a reference or something to substantiate the claim.

Reply

We included the reference Pettersson, L. G. M. In A two-state picture of water and the funnel of life, Modern Problems of the Physics of Liquid Systems, L.A., B.; L., X., Eds. Springer Proceedings in Physics: 2019; pp 3-39 (Ref. 8 in the manuscript).

7) Page 20, line 34. “As pressure increases…there is a fragmentation of LDL domains” needs a reference or data to support this statement. Do they fragment or become less favorable?

Reply

In the text, we used “fragmentation” as a synonym for “less favorable”. Nevertheless, the revised manuscript was modified according to the reviewer’s suggestion.

8) Page 21, lines 44-54. The reason why more thermal energy is required for HDL to LDL fluctuations should result in more LDL domains needs to be given. As stated, it would seem that the result should be less LDL, not more.

Reply

We agree that the sentence was unclear. In the revised version, we clarify the argument stressing that as higher T_c values correspond to a larger amount of HDL motifs (as discussed on page 12 and Figure 2c), we propose that a greater surface charge increases the relative proportion of LDL patches in the hydration water of the particles.

9) Page 23, lines 13-17. “Additionally, we have shown that increasing pH disrupts the tetrahedral organization”. This has not been shown in the present work. It is a speculation.

Reply

In this manuscript, we do not demonstrate directly that increasing pH disrupts the tetrahedral organization. This connection was discussed in ref. 111 of the main manuscript (*Nat. Commun.* 13, 822, **2022**), for instance. We show that increasing pH decreases T_c, and decreasing T_c decreases the relative amount of LDL patches, akin to external pressure on pure water. The sentence was modified in the revised version of the manuscript.

10) Page 23, lines 44-47. “Invaluable for experimentally quantifying HDL/LDL proportions in the hydration water”. The authors have shown interesting differences that they associate (as a hypothesis) with conversion between HDL and LDL, but there has not been any attempt to quantify the fractions. The statement is thus poorly supported by the data that is presented.

Reply

This sentence was removed in the revised version of the manuscript.

11) SI, Point 6. The conversion between HDL and LDL has been experimentally observed in ref 37 (main paper) which could be stronger evidence than Poole et al.

Reply

We agree with the suggestion and the SI was modified accordingly.

12) Figure S9b. Noise histograms (8 classes)? I only see 4 histograms.

Reply

The caption of the submitted version was misleading and is corrected in the revised SI.

Reviewer #2

This is a very interesting work in which authors investigate the variation of the cross-over temperature of water as a function of the particle size used for sensing and as a function of the pH. Authors use upconverting nanoparticles for determining the Brownian velocity and how this Brownian velocity changes with temperature. The crossover temperature (that determines the temperature at which equilibrium between high-density and low-density domains is broken) is determined by analyzing the two linear regimes that are obtained in the plot of Brownian velocity vs temperature. The main conclusion, if I understood well the paper, is that the as the particle size is reduced the presence of high-density domains is reduced and the critical temperature increases as the particle size decreases. I think this conclusion is valuable and reasonable.

We appreciate the reviewer's positive feedback and we have incorporated the suggestions into the revised version of the manuscript.

1. The question here is that authors did not observe any net change of the Brownian velocity with particle size. For sizes between 50 and 100 nm the Brownian velocity seems to be independent on size. This sounds strange for me. Is there any easy explanation for this?

Reply

The question about the dependence of the Brownian velocity on the size of the UCNPs is very pertinent and we thank the reviewer for bringing up this discussion. As noticed by the reviewer, when the size increases from 15 to 64 nm there is a decrease in the Brownian velocity of about 20%, being almost size-independent for larger values (up to 106 nm), **Figure R1a**.

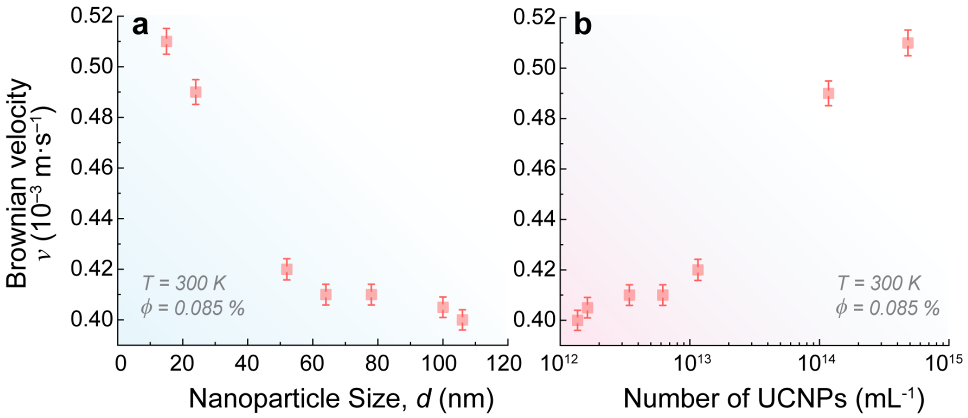

Figure R1. Brownian velocity of the UCNPs in the aqueous nanofluids. The Brownian velocity is represented as a function of **(a)** the particle size and **(b)** the number of UCNPs per unit of volume at 300 K and pH=5.1 (an illustrative temperature).

Due to the same volume fraction used across all colloidal suspensions (0.085%), smaller particle sizes correspond to a greater number of UCNPs in the suspensions (further details provided in the revised SI). Consequently, this results in an increased Brownian velocity of the UCNPs with a higher particle count in suspension. Notably, this effect is more pronounced for smaller UCNPs with sizes of 15 and 24 nm, as illustrated in **Figure R1b**. As depicted in the figure, the number of UCNPs increases by approximately a factor of 4 when the particle size decreases from 106 to 64 nm. However, this increment is far more striking, reaching a factor of 40, when the size decreases from 52 to 15 nm. The observed decrease in Brownian velocity as UCNP size increases is, therefore, attributed to the dependence of Brownian velocity on the number of particles per mL in the suspension (particle-particle interactions). It is noteworthy that these findings align seamlessly with our earlier observations (*Nat. Nanotech.* 11, 851, **2016**).

This discussion (including **Figure R1**) has been incorporated into the revised manuscript and SI.

2. Authors also studied the change of the crossover temperature with the pH. Again I think that the reasoning given by authors is good but then I have some difficulties to understand why the results here obtained do not agree with previous papers also dealing with the variation of the crossover temperature with the pH. By the use of optical tweezers or by using the luminescence of certain complexes it has been found that crossover temperature increases with the pH. But data in Figure 4 seems to indicate that in these experiments the tendency is opposite. I think that this point should be discussed in detail in a revised version.

Reply

We express our gratitude to the reviewer for highlighting this point. We investigated this through literature analysis, as observed on page 18 of the submitted manuscript (and references 80, 82, 83, 86). There is an evident divergence in the findings concerning the crossover temperature dependence with increasing pH. While some studies indicate an increase in the crossover temperature (*e.g.*, *Anal. Bioanal. Chem.* 412, 73, **2020** and *Nano Lett.* 20, 8024, **2020**), another report (*e.g. ACS Omega* 3, 18930, **2018**) aligns with the decrease in the crossover temperature observed by us in the present manuscript. Reference *J. Phys. Chem. C* 122, 14838, **2018** reported a single point for the crossover temperature at pH=5.5, and, thus, no tendency can be discerned. In *Nano Lett.* 20, 8024, **2020**, the authors analyze the escape velocity of UCNPs at pH 5.5 and 7.9 and the observed changes with the crossover temperature are constant within its uncertainty.

Additionally, the way how *"crossover temperature"* is defined differs in the literature. References *J. Phys. Chem. C* 122, 14838, **2018**, *Anal. Bioanal. Chem.* 412, 73, **2020**, and *Nano Lett.* 20, 8024, **2020** presented a series of crossover temperature values extracted from the temperature dependence of pH in a series of pH-buffer solutions and from the temperature dependence of some of the water properties (e.g., thermal conductivity and surface tension). This is difficult to be related to the onset of the LDL-HDL density fluctuations as in our work.

Moreover, the way that pH is changing also differs. In *Nano Lett.* 20, 8024, **2020** and *ACS Omega* 3, 18930, **2018** the adjustment was done as in our work adding small amounts of HCl or NaOH solutions to the aqueous dispersion, while in *Anal. Bioanal. Chem.* 412, 73, **2020** a tris buffer of diverse pH (adjusted by small amounts of HCl or NaOH solutions) is employed.

Finally, we found that different studies report opposite behaviors for the change of surface charge (that we demonstrated in our manuscript that is connected with T_c) with pH. For instance, Barisik et al., (*J. Phys. Chem. C* 118, 1836, **2014**), reported an increase in the magnitude of the surface charge of silica NPs with an increase in pH, while Abbas et al. (*J. Phys. Chem. C* 112, 5715, **2008**), reported a decrease of the surface charge density of metal oxide NPs with an increase in pH. Interestingly, despite the average crossover temperature reported in *Nano Lett.* 20, 8024, **2020** increases with pH (as mentioned above opposite to our reported trend), once their T_c is plotted as a function of the zeta potential the trend is the same as the one reported in our work, i.e., both of them increases as the surface charge increases.

In summary, the comparison between our results and those showing an increase in the crossover temperature with pH is not straightforward. Further investigation is needed to reconcile these differences and clarify the relationship between pH and the crossover temperature. This will be one of the aims of our future studies.
